# Identification and characterisation of non-coding small RNAs in the pathogenic filamentous fungus *Trichophyton rubrum*

**DOI:** 10.1186/1471-2164-14-931

**Published:** 2013-12-30

**Authors:** Tao Liu, Xianwen Ren, Tengfei Xiao, Jian Yang, Xingye Xu, Jie Dong, Lilian Sun, Runsheng Chen, Qi Jin

**Affiliations:** 1 MOH Key Laboratory of Systems Biology of Pathogens, Institute of Pathogen Biology, Chinese Academy of Medical Sciences & Peking Union Medical College, Beijing 100730, China; 2 Bioinformatics Laboratory, Institute of Biophysics, Chinese Academy of Sciences, Beijing 100101, China

## Abstract

**Background:**

Accumulating evidence demonstrates that non-coding RNAs (ncRNAs) are indispensable components of many organisms and play important roles in cellular events, regulation, and development.

**Results:**

Here, we analysed the small non-coding RNA (ncRNA) transcriptome of *Trichophyton rubrum* by constructing and sequencing a cDNA library from conidia and mycelia. We identified 352 ncRNAs and their corresponding genomic loci. These ncRNA candidates included 198 entirely novel ncRNAs and 154 known ncRNAs classified as snRNAs, snoRNAs and other known ncRNAs. Further bioinformatic analysis detected 96 snoRNAs, including 56 snoRNAs that had been annotated in other organisms and 40 novel snoRNAs. All snoRNAs belonged to two major classes—C/D box snoRNAs and H/ACA snoRNAs—and their potential target sites in rRNAs and snRNAs were predicted. To analyse the evolutionary conservation of the ncRNAs in *T. rubrum*, we aligned all 352 ncRNAs to the genomes of six dermatophytes and to the NCBI non-redundant nucleotide database (NT). The results showed that most of the identified snRNAs were conserved in dermatophytes. Of the 352 ncRNAs, 102 also had genomic loci in other dermatophytes, and 27 were dermatophyte-specific.

**Conclusions:**

Our systematic analysis may provide important clues to the function and evolution of ncRNAs in *T. rubrum*. These results also provide important information to complement the current annotation of the *T. rubrum* genome, which primarily comprises protein-coding genes.

## Background

Numerous studies have demonstrated that non-coding RNAs (ncRNAs) are widely expressed in both prokaryotes and eukaryotes [1-4]. Furthermore, the number of ncRNAs substantially increases with the complexity of the organism, whereas the number of protein-coding genes remains relatively static. In bacteria, unicellular eukaryotes, and invertebrates, the coding sequences constitute approximately 95, 30, and 20% of the genomic DNA, respectively. In mammals, open-reading frames only account for approximately 1–2% of the genomes [5-9].

NcRNAs include highly abundant and functionally important RNAs, such as transfer RNA (tRNA) and ribosomal RNA (rRNA), as well as other small, stable RNAs, such as small nuclear RNAs (snRNAs), small nucleolar RNAs (snoRNAs), RNase P and mitochondrial RNA processing (MRP) RNA, signal recognition particle (SRP) RNA, and telomerase RNA. These RNAs have been characterised and are involved in splicing, ribosome biogenesis, translation, and chromosome replication [10,11]. Recent transcriptomic and bioinformatic studies have also identified an increasing number of new ncRNAs whose function has not been validated [12-16]. Hence, the discovery and analysis of ncRNAs has become an important step in our understanding of genomic structure and will expand our knowledge of the function and the regulatory roles of ncRNAs in the cell cycle and development.

In recent years, ncRNAs have been identified using experimental methods and computational predictions in several fungi [3,4,17-22]. A large number of non-coding RNA genes, including 33 box C/D snoRNA genes, have been predicted in the genome of *Schizosaccharomyces pombe*. Functional analyses of 20 Box H/ACA snoRNAs indicated that the snoRNAs evolved in coordination with rRNAs to preserve post-transcriptional modification sites among distant eukaryotes [3,4,20]. A comparative genomics analysis of seven different yeast species identified a substantial number of evolutionarily conserved, structured ncRNAs, suggesting their roles in post-transcriptional regulation [20]. NcRNAs that participate in the cleavage and processing of tRNAs were observed in *Aspergillus fumigatus*[21]. An extensive analysis of snoRNA genes from *Neurospora crassa* indicated a high diversity of post-transcriptional modification guided by snoRNAs in the fungus kingdom [22]. Thus far, the ncRNAs of dermatophytes have not been studied.

*Trichophyton rubrum* is the most common dermatophyte that can infect human keratinised tissue (skin, nails, and, rarely, hair) [23-25]. *T. rubrum* has a 22.5-Mbp haploid nuclear genome consisting of five chromosomes that range in size from 3.0–5.8 Mbp and a 27-kbp circular mitochondrial genome [26]. The Broad Institute has sequenced the *T. rubrum* genome and predicted more than 8,700 protein-coding genes. However, apart from rRNAs and tRNAs, no other ncRNAs have been annotated and characterised within the *T. rubrum* genome [26]. In the present study, we constructed an ncRNA library (ranging from 70–500 nt) and identified ncRNAs in *T. rubrum* using an RNA-Seq method. A total of 352 ncRNA candidates were characterised, including 198 entirely novel ncRNAs and 154 known ncRNAs. We also analysed the sequence conservation, and genomic location of these ncRNAs in six other dermatophytes. Our results may guide further studies of the important roles of ncRNA in *T. rubrum* and provide important complementary information to the annotation of the *T. rubrum* genome.

## Results

### *Identification of ncRNA candidates in* T. rubrum

**Figure 1:**
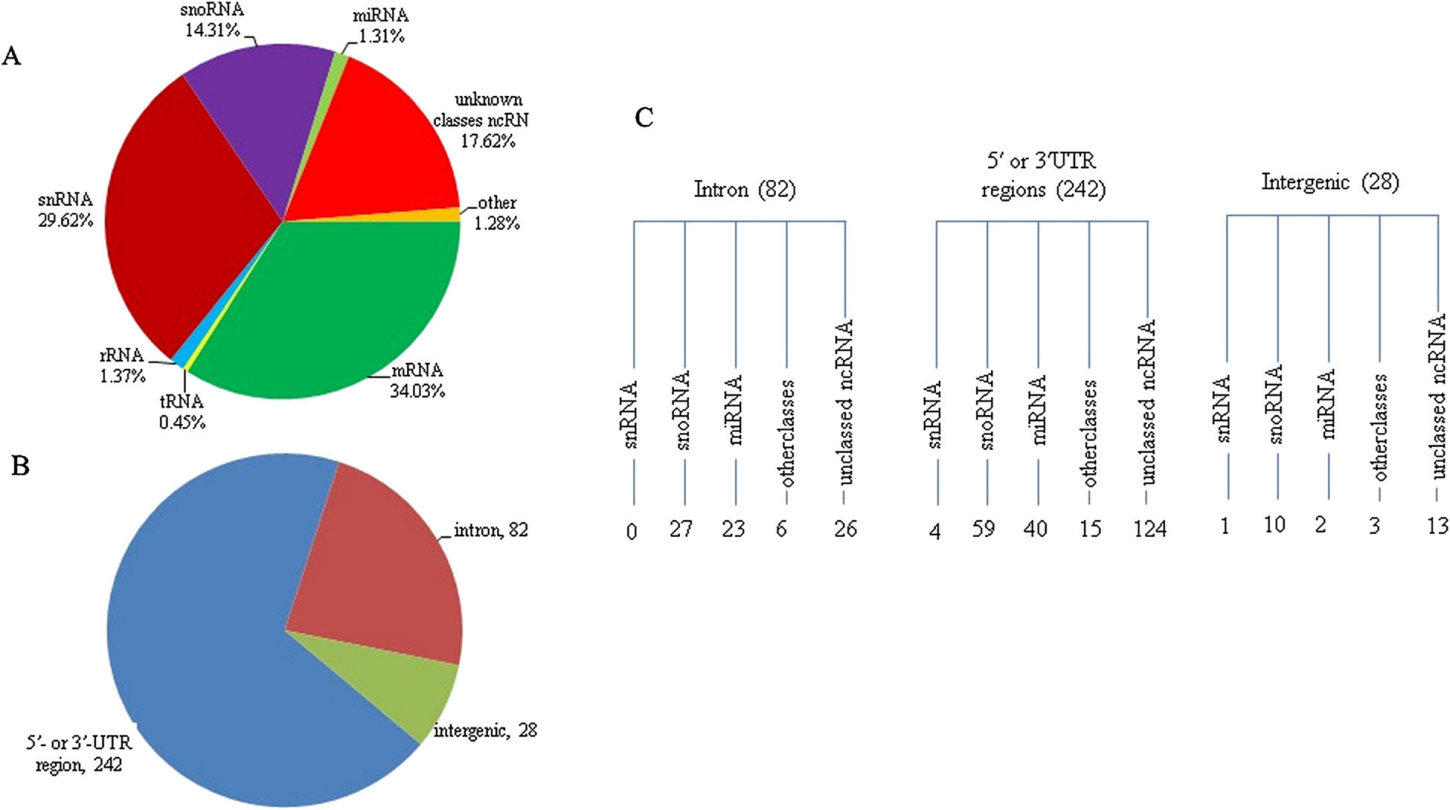
**Detection of ncRNA candidates in T. rubrum by sequencing a size-fractionated cDNA library. (A)** The distribution of 87,601 reads from the constructed small cDNA library of *T. rubrum* in different RNA classes. **(B)** The numbers of ncRNAs from different regions in the *T. rubrum* genome. **(C)** The number of different classes of ncRNAs are displayed in brackets.

To obtain a global view of ncRNAs in *T. rubrum*, we extracted total RNA from the conidia and mycelia phases and generated a small RNA cDNA library with size-fractionated total RNA ranging in size from 70–500 bp. After sequencing on the 454/Roche sequencing platform, a total of 87,601 reads were obtained and mapped to the *T. rubrum* genome. Next, the reads that mapped to the same genomic loci were clustered, resulting in 4,432 unique contigs. After removing the coding RNA and matches to tRNAs and rRNAs, the remaining 352 clusters (corresponding to 56,550 reads) were considered ncRNA candidates. Of these candidates, 154 were predicted to align with Rfam sequences and the remaining 196 were novel ncRNA candidates (Figure 1; for detailed information, see Additional file 1: Table S1).

### Characteristics of ncRNA candidates

**Figure 2:**
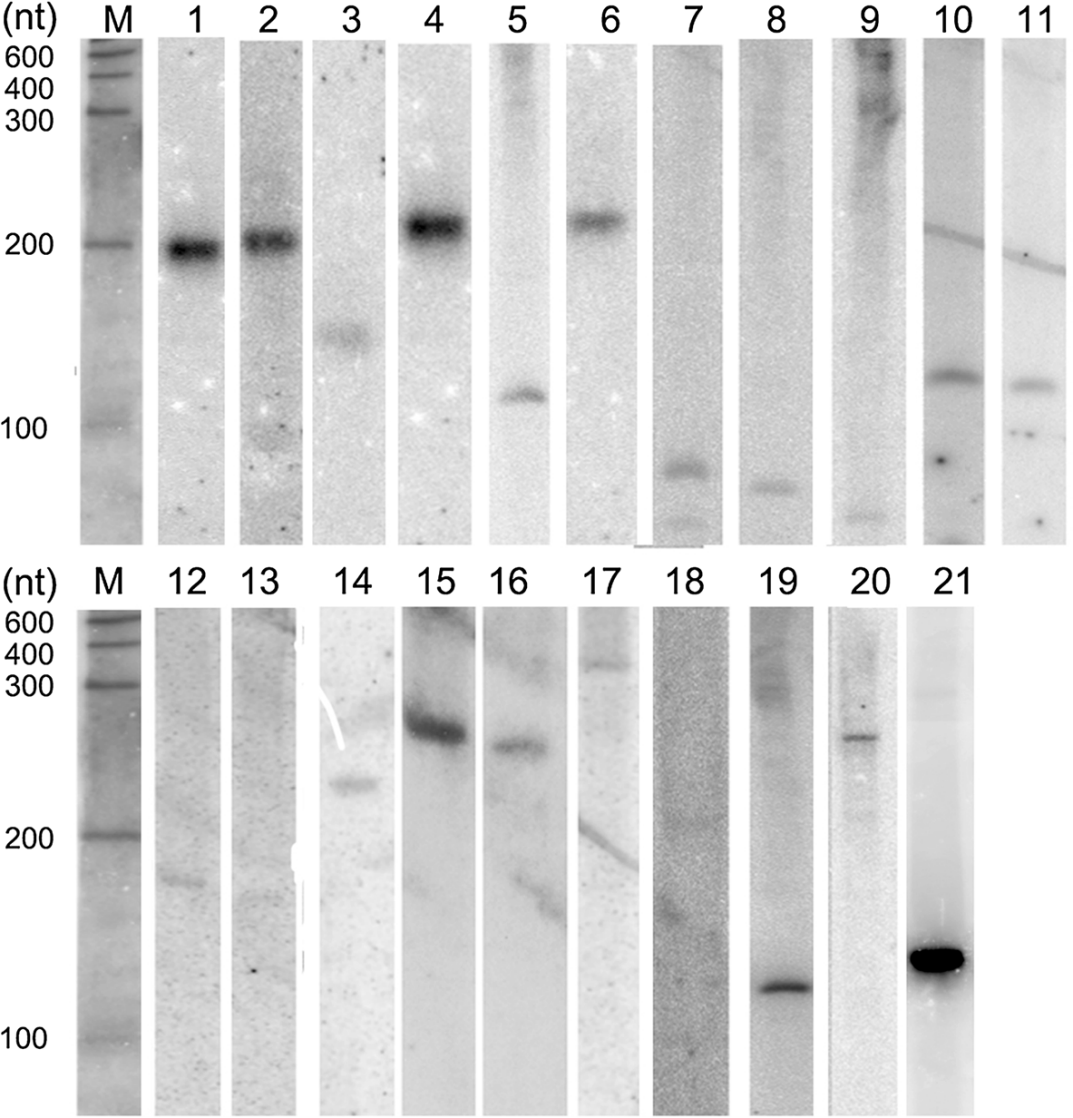
**Northern blotting analysis of *T. rubrum *ncRNA candidates.** M. RiboRuler Low Range RNA Ladder (Fermentas), 1. snRNA U1, 2. snRNA U2, 3. snRNA U4, 4. snRNA U5, 5. snRNA U6, 6. Trnc_2843, 7. Trnc_3589, 8.Trnc_369, 9. Trnc_1414, 10. Trnc_293, 11. Trnc_305, 12. Trnc_1472, 13. Trnc_961, 14. Trnc_608, 15. Trnc_4262, 16. Trnc_1437, 17. Trnc_2618, 18. Trnc_3096, 19. Trnc_1686, 20. TRnc2844, and 21. 5.8S rRNA. The lengths and other information describing the ncRNAs from the northern blotting analysis are shown in Additional file 1: Table S1.

Of the 352 identified ncRNA candidates, 234 mapped to loci within 1 kb of the closest coding gene, implying a possible functional relationship. Some of the ncRNA clusters located in the immediate vicinity of a protein-coding region might be processed from the 5′- or 3′-UTR of the corresponding mRNA. Among the 352 ncRNA clusters, 82 were intronic and 29 corresponded to non-annotated intergenic regions of the *T. rubrum* genome (Figure 1). To verify the expression and sizes of candidate ncRNAs, we selected the spliceosomal snRNAs U1, U2, U4, U5, and U6 and 15 randomly selected novel ncRNA candidates to use in northern hybridisation. The results are shown in Figure 2.

### snRNA candidates

The spliceosome contains five essential small nuclear RNAs (snRNAs)—U1, U2, U4, U5, and U6—that are essential components for assembling the spliceosome and accomplishing the intricate task of intron removal from newly synthesised eukaryotic RNAs [17,18,27]. Here, we identified the genomic loci of snRNAs U1, U2, U5, and U6, each of which exhibited a unique genomic location. U5 and U6 were the most abundant snRNAs among our data, found in 15,583 and 9,034 reads, respectively. The expression of U2 and U4 was lower than the other snRNA candidates; we found only 163 reads of U2 and 146 reads of U4. These results are in agreement with those of the small ncRNA transcriptome analysis of another filamentous fungus, *A. fumigatus*[21,28]. U4 was not initially identified in our data. To find the U4 genomic locus in *T. rubrum*, we downloaded the U4 sequences of *A. fumigatus*, *A. oryzae*, and *A. niger* from Rfam to use as query sequences to search for homologues in the *T. rubrum* genome using BLASTn. One genomic locus was identified. Corresponding reads assigned to the same locus had been sequenced and clustered in our data but had been eliminated because the percentage of ORF in the cluster was greater than 80%.

We aligned the *T. rubrum* snRNA U1, U2, U4, U5, and U6 candidates to the genomes of six *T. rubrum*-related dermatophytes to predict the homologues in these genomes by BLASTn. The homologues were compared using the multiple sequence alignment software ClustalW2, revealing that all snRNAs were highly conserved in these seven dermatophytes (Table 1). High variance was observed among the sequences and lengths of these snRNAs in *T. rubrum* and their homologues in other fungi; however, these snRNAs were conserved at the secondary structure level, with conserved regions in the hairpin loops (Additional file 2: Figure S2). These results correspond with previous reports on *A. fumigatus*[21].

**Table 1:** **Conservation level of snRNAs in *T. rubrum *** **and related dermatophytes**

			**Genome location**					
**Name**	**Genes**	**Len**^**a**^	**Chromosome**	**Start**	**End**	**Position**	**Conserved in dermatophytes (% sequence identity)**	**Accession**
Trnc_3904	U1	196	supercont2.8	159538	159733	5′UTR	*M. gypseum* (98%), *M. canis* (98%), *A. benhamiae* (100%)	KC353306
Trnc_774	U2	201	supercont2.1	3545014	3545214	3′UTR	*T. tonsurans* (98%), *T. equinum* (98%), *M. gypseum* (97%), *T. verrucosum* (99%), *M. canis* (96%), *A. benhamiae* (99%)	KC353051
Trnc_1437	U4	264	supercont2.17	13253	15593	Intergenic	*T. tonsurans* (100%), *T. equinum* (100%), *M. gypseum* (99%), *A. benhamiae* (100%)	KC353100
Trnc_681	U5	211	supercont2.1	3061687	3061897	5′UTR	*T. tonsurans* (92%), *T. equinum* (92%), *M. gypseum* (95%), *T. verrucosum* (93%), *M. canis* (91%), *A. benhamiae* (100%)	KC353044
Trnc_1782	U6	104	supercont2.2	1801544	1801647	3′UTR	*T. tonsurans* (100%), *M. gypseum* (100%), *M. canis* (99%), *A. benhamiae* (100%)	KC353131

Len^a^: the cDNA length of the snRNA. Conserved in dermatophytes (% sequence identity): the sequence identity of homologous snRNAs in other dermatophytes compared to *T. rubrum*; Accession is the accession number in GenBank.

### snoRNAs

In eukaryotic cells, two major classes of small nucleolar ncRNA (snoRNA) have been identified: C/D box snoRNAs, which are involved in the 20-O-methylation of ribosomal, spliceosomal, and transfer RNAs (the latter in Archaea only), and H/ACA snoRNAs, which guide pseudouridylation in these RNA species [29,30].

To predict the two classes of snoRNAs and their putative targets in our data, we used the Snoscan and SnoGPS programs, defining the potential target sequences as the 5.8S, 18S, and 25S rRNAs of *T. rubrum* and all snRNAs identified in our data [17,18]. We identified 96 snoRNAs, including 58C/D box snoRNAs (46 had homologues in other organisms) and 38H/ACA snoRNAs (nine had homologues in other organisms). We identified 37C/D box snoRNAs as putative targets, most of which were predicted to guide methylation of 18S and 25S rRNAs. We also identified five C/D box snoRNAs (TRnc_801, TRnc_3573, TRnc_4113, TRnc_1272, and TRnc_1271) that were predicted to guide the methylation of snRNAs U1, U2, and U5. Of the 37C/D box snoRNAs, 22 had different modification sites in target rRNA or snRNA sequences. No rRNA or snRNA targets were identified in the remaining 21C/D box snoRNAs (Table 2). Additionally, the 30 identified H/ACA box snoRNAs were identified as guiding the pseudouridylation of 45 sites in rRNAs (Table 3. Detail information about potential base-paring between H/ACA box snoRNAs and rRNA shown in Additional file 3: Figure S3), whereas no pseudouridine sites were predicted on any snRNAs.

**Table 2:** **C/D box snoRNA candidates identified in *T. rubrum***

		**Genome position**				**Homologues**			
**Name**	**Len**^**a**^	**Chromosome**	**Start**	**End**	**Location**	**Accession**^**1**^	**Genes**	**Putative target(s)**	**Accession**^**2**^
TRnc_1010	87	supercont2.10	749220	749306	3′UTR	RF00477	snosnR66		KC353070
TRnc_1157	95	supercont2.11	539262	539356	Intron	RF00093	SNORD18, U18	25S: Am651, Gm654; 18S: Am1159	KC353075
TRnc_1271	242	supercont2.12	280437	280196	Intron	RF01152	sR1	25S: Am2268, Am3277,Cm964,Cm961;U5: Cm103; 18S: Am1540	KC353083
TRnc_1272	265	supercont2.12	280712	280448	Intron	RF01152	sR1	25S: Cm964, Cm961;18S: Um604; U5: Cm103	KC353084
TRnc_1299	109	supercont2.13	24837	24729	Intron	RF00593	snoU83B		KC353086
TRnc_1359	97	supercont2.14	159345	159441	Intron	RF00475	snosnR69	25S: Cm3322	KC353090
TRnc_1366	215	supercont2.14	179253	179467	3′UTR	RF01152	sR1		KC353091
TRnc_1449	234	supercont2.17	97081	97314	5′UTR	RF01191	SNORD121A	18S: Cm673, Gm234	KC353101
TRnc_1560	77	supercont2.2	546818	546894	3′UTR	RF01139	sR2		KC353110
TRnc_1603	358	supercont2.2	766347	766704	3′UTR	RF00345	snoR1		KC353115
TRnc_1709	154	supercont2.2	1400380	1400533	5′UTR	RF01193	snoR20a		KC353124
TRnc_1825	309	supercont2.2	1958330	1958022	3′UTR			25S: Um2301; Um769	KC353137
TRnc_1841	143	supercont2.2	2090171	2090313	3′UTR	RF01144	sR17		KC353138
TRnc_2011	127	supercont2.3	74633	74759	3′UTR	RF00441	snoZ242		KC353147
TRnc_2018	306	supercont2.3	117035	116730	3′UTR			18S: Um628	KC353149
TRnc_2027	96	supercont2.3	166668	166763	Intron	RF01281	snoR35		KC353150
TRnc_2179	431	supercont2.3	961995	961565	Intergenic			25S: Um413	KC353160
TRnc_2265	87	supercont2.3	1276133	1276219	Intron	RF01197	snR39	25S: Gm808	KC353164
TRnc_2283	233	supercont2.3	1301587	1301819	5′UTR			18S: Am1105; 25S: Am499, Am1453	KC353165
TRnc_2405	317	supercont2.3	1975149	1975465	3′UTR			25S: Gm1738	KC353175
TRnc_2419	204	supercont2.3	2045771	2045974	5′UTR	RF01125	sR4	18S: Am350, Gm698, Cm701;25S: Gm215, Cm3127	KC353177
TRnc_2421	182	supercont2.3	2046135	2046316	5′UTR	RF00016	SNORD14, U14	18S: Um50, Cm379;25S: Cm2352	KC353178
TRnc_2498	172	supercont2.3	2451919	2452090	5′UTR	RF00527			KC353188
TRnc_2545	119	supercont2.3	2657688	2657806	3′UTR	RF01188	snR56	18S: Gm1389,Am385	KC353195
TRnc_2569	192	supercont2.3	2759920	2759729	Intron	RF01297	sR40		KC353197
TRnc_2594	143	supercont2.3	2859175	2859033	Intron	RF01305	sR51		KC353199
TRnc_2691	158	supercont2.4	233433	233276	Intergenic			5.8S: Gm87	KC353216
TRnc_2782	128	supercont2.4	669565	669438	5′UTR	RF00630	P26	18S: Cm534; 25S: Cm1583, Cm1196, Cm3233	KC353223
TRnc_2936	246	supercont2.4	1403883	1403638	Intron	RF00312	snoZ206	25S: Gm1378	KC353235
TRnc_3227	139	supercont2.5	625518	625380	Intron	RF00594	SNORD86, U86		KC353256
TRnc_3297	138	supercont2.5	896392	896529	3′UTR	RF00610	SNORD110		KC353262
TRnc_338	135	supercont2.1	1643180	1643314	Intron	RF01223	snR13	25S: Am2267	KC353022
TRnc_3425	202	supercont2.6	22581	22782	3′UTR			25S: Gm911	KC353267
TRnc_3426	98	supercont2.6	23000	23097	3′UTR				KC353268
TRnc_3438	173	supercont2.6	91295	91467	5′UTR	RF01291	snoU97, SNORD97		KC353269
TRnc_3573	95	supercont2.6	964586	964680	Intron	RF00530	snoMe28S-Cm2645	25S: Cm2324, Um2867; U2: Um43	KC353276
TRnc_3654	191	supercont2.7	14823	15013	3′UTR	RF01140	sR20	18S: Gm832	KC353284
TRnc_3667	191	supercont2.7	59063	59253	3′UTR	RF00529	snoMe28S-Am2589		KC353285
TRnc_3778	101	supercont2.7	777627	777727	5′UTR	RF00471	snosnR48, snr46	18S: Am721; 25S: Gm2780; Am2243	KC353293
TRnc_3833	109	supercont2.7	1124537	1124429	3′UTR	RF01273	sR34		KC353299
TRnc_3855	288	supercont2.7	1281447	1281734	Intron	RF01127	sR42		KC353305
TRnc_3911	80	supercont2.8	194694	194773	Intron	RF00213	snoR38	25S: Gm2799	KC353308
TRnc_4113	681	supercont2.8	1152047	1152727	Intron	RF01274	sR45	25S: Cm1856,Cm1673; 18S: Am833; U2: Am155	KC353324
TRnc_415	103	supercont2.1	1918108	1918210	Intron	RF01121	Sr38		KC353027
TRnc_4250	192	supercont2.9	658585	658394	3′UTR			18S: Cm373	KC353339
TRnc_4259	104	supercont2.9	693331	693434	5′UTR	RF00276	SNORD52, U52	25S: Um2408	KC353340
TRnc_4260	95	supercont2.9	695194	695288	Intergenic	RF01178	snoR77Y,snR77	18S: Um565, Am564	KC353341
TRnc_4261	138	supercont2.9	695445	695582	Intergenic	RF01209	snR76	18S: Cm1674;25S: Cm2184, Am2266, Cm3294, Cm1758	KC353342
TRnc_4262	273	supercont2.9	695588	695860	Intergenic	RF01185	snR75, U15	25S: Gm2275	KC353343
TRnc_4263	157	supercont2.9	695917	696073	Intergenic	RF00086	SNORD27, U27, snR74	25S: Cm1179	KC353344
TRnc_4264	88	supercont2.9	696179	696266	5′UTR	RF01207	snR73,U35	25S: Cm3333	KC353345
TRnc_4267	100	supercont2.9	703004	703103	3′UTR			18S: Um525, Gm527	KC353346
TRnc_4316	97	supercont2.9	861468	861372	5′UTR	RF01223	snR13		KC353347
TRnc_4336	162	supercont2.9	996654	996493	Intron			18S: Gm1089	KC353348
TRnc_608	234	supercont2.1	2701229	2701462	3′UTR	RF01202	sn2991	5.8S: Cm137	KC353041
TRnc_640	129	supercont2.1	2869815	2869687	3′UTR	RF00300	snoZ221		KC353043
TRnc_801	488	supercont2.1	3681448	3681935	3′UTR	RF00012	U3	18S: Um418; 25S: Cm1363, Cm1633, Cm1983, Cm3165; U1: Cm45	KC353053
TRnc_821	210	supercont2.1	3768831	3768622	Intergenic			18S: Cm1301,25S: Cm880	KC353055
TRnc_985	153	supercont2.10	686423	686575	Intron	RF00494	snoU2_19		KC353066

Name: the C/D box snoRNAs were numbered according to the order of identification. Len^a^: the cDNA length of the snoRNA. Homologues: homologues in Rfam or other organisms. Accession^1^ is the accession number in Rfam; Accession^2^ is the accession number in GenBank; Genes are homologous gene names in other organisms [19-22]. Putative target(s): the predicted modified nucleotides within rRNAs or snRNAs using the Snoscan package.

**Table 3:** **H/ACA box snoRNA candidates identified in *T. rubrum***

		**Genome location**				**Homologues**			
**Name**	**Len**^**a**^	**Chromosome**	**Start**	**End**	**Position**	**Accession**^**1**^	**Genes**	**Putative target**	**Accession**^**2**^
Trnc_1355	371	supercont2.14	142837	142467	5′UTR			18S-Ψ1434	KC353088
Trnc_1370	133	supercont2.14	187697	187565	5′UTR	RF01134	sR30		KC353092
Trnc_203	308	supercont2.1	996485	996178	5′UTR			18S-Ψ803	KC353013
Trnc_2045	228	supercont2.3	296293	296520	5′UTR			25S-Ψ2867,18S-Ψ489	KC353151
Trnc_2579	349	supercont2.3	2792710	2793058	5′UTR			18S-Ψ611	KC353198
Trnc_2999	290	supercont2.4	1720998	1721287	5′UTR			25S-Ψ2135	KC353240
Trnc_3005	214	supercont2.4	1748930	1749143	5′UTR			25S-Ψ1081	KC353241
Trnc_3218	332	supercont2.5	584674	585005	5′UTR			18S-Ψ573,25S-Ψ681,25S-Ψ2635	KC353255
Trnc_3509	433	supercont2.6	608530	608098	5′UTR			25S-Ψ2545,25S-Ψ1671	KC353274
Trnc_5	289	supercont2.1	19982	20270	5′UTR			25S-Ψ2329	KC352999
Trnc_910	468	supercont2.10	343107	343574	5′UTR			18S-Ψ12	KC353060
Trnc_1407	234	supercont2.16	54707	54474	3′UTR			25S-Ψ1155	KC353095
Trnc_1472	188	supercont2.2	69663	69850	3′UTR	RF01258	snR10		KC353105
Trnc_1776	326	supercont2.2	1789188	1788863	3′UTR	RF01231	snoR74	18S-Ψ1593,18S-Ψ412	KC353129
Trnc_1893	344	supercont2.2	2393882	2393539	3′UTR			25S-Ψ312	KC353142
Trnc_2452	323	supercont2.3	2170039	2169717	3′UTR			25S-Ψ2650	KC353184
Trnc_2596	324	supercont2.3	2882125	2881802	3′UTR			18S-Ψ1336	KC353200
Trnc_2843	225	supercont2.4	976176	976400	3′UTR	RF01251	snR3	25S-Ψ2120,25S-Ψ2251	KC353227
Trnc_3023	182	supercont2.4	1839416	1839597	3′UTR			25S-Ψ759,25S-Ψ1558,25S-Ψ520	KC353242
Trnc_3387	226	supercont2.5	1472165	1472390	3′UTR			18S-Ψ565,25S-Ψ2404	KC353265
Trnc_3741	180	supercont2.7	491853	492032	3′UTR	RF01247	snR32		KC353292
Trnc_4007	239	supercont2.8	722404	722166	3′UTR			18S-Ψ1344	KC353317
Trnc_64	306	supercont2.1	267027	267332	3′UTR			25S-Ψ2714	KC353002
Trnc_817	188	supercont2.1	3719705	3719892	3′UTR			18S-Ψ267,18S-Ψ1697	KC353054
Trnc_920	310	supercont2.10	389299	389608	3′UTR			25S-Ψ116,18S-Ψ1213	KC353061
Trnc_1698	360	supercont2.2	1345609	1345968	Intron			18S-Ψ1026	KC353122
Trnc_2075	96	supercont2.3	425677	425772	Intron	RF00405	SNORA44		KC353153
Trnc_2172	126	supercont2.3	922150	922025	Intron	RF00406	SNORA42		KC353159
Trnc_2443	106	supercont2.3	2090244	2090349	Intron	RF00428	SNORA38		KC353182
Trnc_2531	75	supercont2.3	2617075	2617001	Intron	RF00415	SNORA30		KC353194
Trnc_2606	280	supercont2.36	2106	2385	Intergenic			25S-Ψ1054	KC353202
Trnc_2618	322	supercont2.36	8062	8383	Intergenic			25S-Ψ1062	KC353205
Trnc_2621	406	supercont2.36	8934	9339	Intergenic			25S-Ψ1689	KC353206
Trnc_2636	203	supercont2.36	19276	19478	Intergenic			18S-Ψ217,25S-Ψ1890	KC353210
Trnc_2898	393	supercont2.4	1199167	1198775	Intron			25S-Ψ1718,25S-Ψ36	KC353231
Trnc_3585	281	supercont2.6	1065274	1064994	Intron			18S-Ψ867,25S-Ψ111	KC353278
Trnc_4006	251	supercont2.8	710950	711200	Intron	RF01263	snR191	18S-Ψ935,25S-Ψ1239,25-Ψ2245	KC353316

Name: the H/ACA box snoRNAs were numbered according to the order of identification. Len^a^: the cDNA length of the snoRNA. Homologues: homologues in Rfam or other organisms. Accession^1^ is the accession number in Rfam; Accession^2^ is the accession number in GenBank; Genes are homologous gene names in other organisms [19-22]. Putative target(s): the predicted modified nucleotides within rRNAs using SnoGPS package.

### *Other types of ncRNA in* T. rubrum

We also identified 51 other ncRNA genomic loci, such as pri-miRNAs or pre-miRNAs, RNAse MRP, and telomerase RNA. miRNAs related transcriptional loci were the most widely distributed ncRNAs in the *T. rubrum* genome; for example, the mir-598 miRNA family had 13 transcriptional regions and mir-533 had eight. In our data, these miRNA homologies of ncRNAs, which varied from 70–270 bp, were much longer than the lengths of mature miRNAs (18–25 bp), they may be pri- or pre-miRNAs candidates.

### *Evolutionary conservation of the ncRNAs in* T. rubrum

To analyse the evolutionary conservation of ncRNAs in *T. rubrum*, we used BLASTn to align the sequences of all 352 ncRNAs to the genomes of six related dermatophytes: *T. equinum*, *T. tonsurans*, *T. verrucosum*, *A. benhamiae*, *M. gypseum*, and *M. canis*. The loci of 102 of these sncRNAs were also identified in all six genomes (Additional file 4: Table S4). We found that the sequences of these sncRNAs were highly conserved, with sequence identities above 85%. Of the 352 ncRNAs, ten had no hits in other genomes and might be specifically expressed in *T. rubrum* (Table 4). To further analyse the conserved ncRNAs in dermatophytes, we employed BLASTn to align all of the sncRNAs with the NCBI non-redundant nucleotide database (NT) after excluding *Arthrodermataceae*. These BLASTn results were processed by MEGAN4, which placed each ncRNA sequence in a node in the NCBI taxonomy [31].

**Table 4:** **The ncRNA candidates specifically expressed in *T. rubrum***

				**Genome location**				
**Name**	**Class**	**Reads**	**Len**^**a**^	**Supercontig**	**Start**	**End**	**Position**	**Accession**
Trnc_20		1	94	supercont2.1	48466	48559	3′UTR	KC353103
Trnc_1456		1	94	supercont2.18	53193	53100	3′UTR	KC353000
Trnc_2606	snoRNA;H/ACA-box	2	280	supercont2.36	2106	2385	Intergenic	KC353202
Trnc_2609		4	255	supercont2.36	4048	4302	Intergenic	KC353203
Trnc_2621	snoRNA;H/ACA-box	97	406	supercont2.36	8934	9339	Intergenic	KC353206
Trnc_2633		297	597	supercont2.36	17132	17728	Intergenic	KC353209
Trnc_2636	snoRNA;H/ACA-box	1	203	supercont2.36	19276	19478	Intergenic	KC353210
Trnc_2640		2	71	supercont2.36	21309	21379	Intergenic	KC353211
Trnc_2649		2	79	supercont2.36	23976	24054	Intergenic	KC353212
Trnc_3096		1	201	supercont2.4	2153644	2153444	3′UTR	KC353244

Len^a^: the cDNA length of the ncRNAs; Accession is the accession number in GenBank. This table shows the lengths and genomic loci of ten ncRNAs that might be specifically expressed in *T. rubrum*. These ncRNAs have no hits assigned to the NCBI NT database using BLASTn.

**Figure 3:**
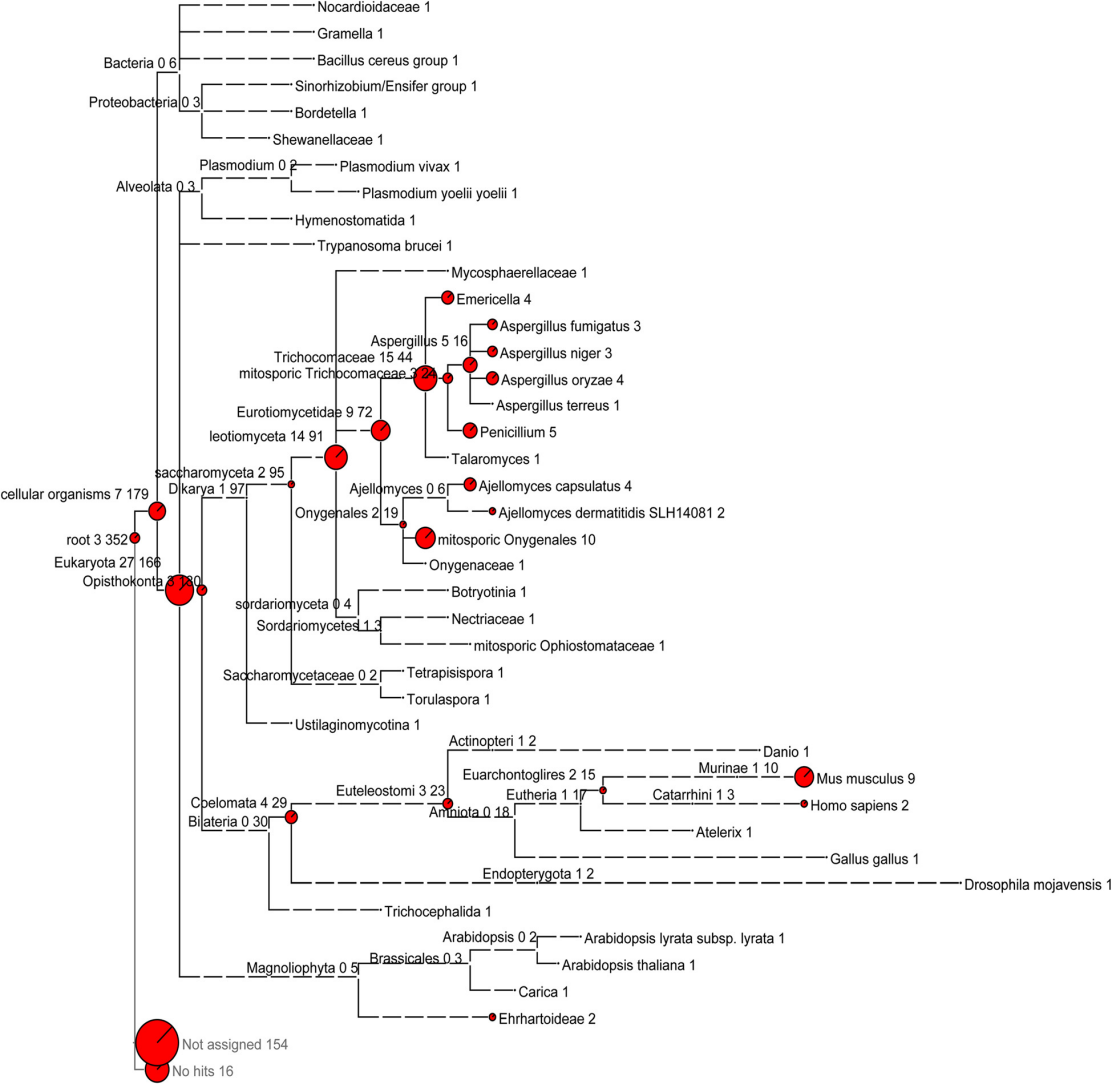
**MEGAN phylogenetic analysis of *T. rubrum *ncRNA candidates.** A MEGAN tree with the taxonomic affiliation of 352 ncRNAs that were identified by BLASTN of all sequences in NT after excluding *Arthrodermataceae* according to NCBI taxonomy. Each circle of the MEGAN tree represents a taxon in the NCBI taxonomy database and is labelled by its name and the number of snRNAs that were assigned to the taxon and not to a subtaxon. The size of the circles represents the number of ncRNAs.

As shown in Figure 3, a total of 179 ncRNA sequences were classified under cellular organisms, with 166 clustered to the Eukaryota node (approximately 47.2% of the total 352 ncRNAs). Of these ncRNAs, 97 were assigned to Fungi, indicating that these ncRNAs were conserved in fungi; all snRNAs were assigned to this node. Of the ncRNAs under the Fungi taxonomic level, 16 and 44 were assigned to *Onygenales* and *Trichocomaceae*, respectively, supporting the close relationship between the dermatophytes and the fungi in these families. Seventy-three ncRNAs were assigned to phyla distantly related to fungi, including three assigned to the root, seven to cellular organisms, 27 to the Eukaryota node, 30 under Bilateria, and six under Bacteria. These results suggest that some ancient ncRNAs are preserved in *T. rubrum*.

Apart from the classified ncRNAs, the remaining 170 ncRNA candidates had no significant similarity to any nucleotide sequence in NT, including 154 unassigned ncRNAs and 16 ncRNAs with no hits. Of these unclassified ncRNAs, 27 existed in and were conserved in all six dermatophytes, indicating that these 27 ncRNAs were dermatophyte-specific ncRNAs (Table 5).

**Table 5:** The ncRNA candidates specifically expressed in dermatophytes

			**Genome location**				
**Name**	**Len**^**a**^	**Reads**	**Chromosome**	**Start**	**End**	**Position**	**Accession**
Trnc_817	188	323	supercont2.1	3719705	3719892	3′UTR	KC353054
Trnc_733	174	1	supercont2.1	3371115	3371288	3′UTR	KC353049
Trnc_2676	156	2	supercont2.4	110438	110593	3′UTR	KC353213
Trnc_3999	178	5	supercont2.8	672734	672557	3′UTR	KC353314
Trnc_1167	177	1	supercont2.11	544895	545071	3′UTR	KC353076
Trnc_2448	161	1	supercont2.3	2123075	2122915	5′UTR	KC353183
Trnc_4219	104	1	supercont2.9	449429	449532	5′UTR	KC353335
Trnc_956	241	2	supercont2.10	559285	559525	5′UTR	KC353063
Trnc_305	97	579	supercont2.1	1515685	1515781	Intron	KC353018
Trnc_500	203	1	supercont2.1	2298649	2298447	Intron	KC353035
Trnc_1792	251	1	supercont2.2	1856556	1856806	Intron	KC353132

Len^a^: the cDNA length of the ncRNAs; Accession is the accession number in GenBank. This table shows the lengths and genomic loci of ten sncRNAs that might be specifically expressed in dermatophytes. These ncRNAs were conserved in all six dermatophytes but have no homologues in NT.

## Discussion

RNA is emerging as a central player in cellular regulation, with active roles in multiple regulatory layers, including transcription, RNA maturation, RNA modification, and translational regulation [32]. Recent studies have revealed an unexpected complexity of regulatory RNAs, even in bacteria [2,33]. In the present study, we first used an RNA-Seq method to analyse the ncRNAs in the genome of the dermatophyte fungus *T. rubrum*. We identified 352 sncRNA candidates, including snRNAs, snoRNAs, miRNAs, and other types of ncRNAs; 196 novel ncRNAs were predicted. We further confirmed the genomic loci of these ncRNAs in *T. rubrum*. This work provides an important complement to the current annotation of the *T. rubrum* genome, which is currently comprised primarily of protein-coding genes.

Five types of snRNAs (U1, U2, U4, U5, and U6) were identified, and their secondary structures were predicted by RNAfold [27]. We found these snRNAs to be highly conserved among dermatophytes. We also detected 96 snoRNAs, including 55 that were annotated in other organisms and 41 that were novel snoRNAs. Using the Snoscan and snoGPS programs, we bioinformatically identified their potential target sites on rRNAs and snRNAs. miRNAs have been previously reported in some fungi, such as *S. pombe*, but have not been found in *A. fumigatus*[21,34]. In our data, we detected 68 genomic loci corresponding to 12 miRNA families; the lengths of these ncRNAs varied from 80–270 bp, suggesting that they were pri-miRNAs or pre-miRNAs [35]. To analyse the evolutionary conservation of ncRNAs, we aligned the 352 snRNAs to six other dermatophyte genomes and the NT database; we found 27 dermatophyte-specific ncRNAs and 11 *T. rubrum*-specific ncRNAs.

## Conclusions

In this study, sequences for ncRNAs were obtained in *T.rubrum* and characterized by sequence comparison to know ncRNAs in other organisms, some of which were presumably functionally characterized in other work. This will prove to be a valuable resource but real understanding of regulatory mechanisms will come from followon work from this strong beginning.

## Methods

### Strain and culture conditions

The *T. rubrum* strain BMU01672 was grown on potato glucose agar (Difco) at 28°C for ten days to produce conidia. The conidia were isolated as previously reported, introduced into YPD medium (2% dextrose, 2% Bacto-Peptone, and 1% yeast extract), and incubated at 28°C with constant shaking at 200 rpm (Innova 4230 Refrigerated Incubator Shaker; New Brunswick Scientific, Edison NJ) [36]. After culture, the mycelia were harvested and ground to a powder in liquid nitrogen for RNA extraction.

### RNA extraction and cDNA library construction

Total RNA was extracted from conidia and mycelia using the RNeasy Plant Mini Kit (Qiagen, Hilden, Germany) according to the manufacturer’s instructions. Same amount of total RNA from conidia and mycelia was mixed and pooled on a denaturing 8% polyacrylamide gel [7 M urea and 1× TBE buffer (90 mM Tris, 64.6 mM boric acid, 2.5 mM EDTA, pH 8.3)]. We collected gel bands containing RNAs of 70–500 bp, excluding the 5.8S rRNA band. RNAs were passively eluted and then ethanol-precipitated. RNA size and concentration were quantified with the Agilent 2100 Bioanalyser and the Agilent RNA 6000 Pico Kit according to the manufacturer’s protocols. The fractionated RNA was dephosphorylated with FastAP (Fermentas) and ligated to the 3′-adaptor oligonucleotide (UUUUGACCACGGTACCCAG, RNA is underlined) by T4 RNA ligase (Promega). Subsequently, the RNA was reverse transcribed using oligo 3RT (CTGGGTACCGTGGTCAAA) and converted into double-stranded cDNA with a SuperScript Double-Stranded cDNA Synthesis Kit (Invitrogen). The ds-cDNA was purified using the MinElute Reaction Cleanup Kit (Qiagen) according to the manufacturer’s protocol.

### 454/Roche sequencing and data bioinformatic analysis

For 454/Roche sequencing, approximately 5 μg of the size-fractionated cDNA sample (70–500 bp) was blunted. The pieces were then ligated with short adaptors prior to amplification and sequencing. The sequencing run was performed using the method of Margulies *et al.*[37].

After 454 sequencing, the 5′ and 3′ adaptors were removed from the reads. Genome data for *T. rubrum* and six related dermatophytes (*Trichophyton equinum, Trichophyton tonsurans, Trichophyton verrucosum, Arthroderma benhamiae, Microsporum gypseum*, and *Microsporum canis*) were downloaded from the Broad Institute web site (http://www.broadinstitute.org/annotation/genome/dermatophyte_comparative/MultiDownloads.html).

The high-quality reads were mapped to the genome using BLAST (version 2.2.22) (Eval < 1e − 5). Then, reads that were 80% mapped to the genome were clustered according to their genomic position and assembled into contigs according to the genomic sequence at the corresponding loci. The ORFs in the contigs were predicted using getorf in the EMBOSS program (version 6.3.1). Contigs with less than 80% ORF were aligned to TrED EST sequences and the NCBI non-redundant protein sequence database (NR) [38,39]. The clusters with no hits in the TrED EST sequences and NR were used for the following steps: (1) alignment to non-coding RNA sequences with rRNA sequences downloaded from Rfam and GenBank [40], (2) identification of tRNAs with tRNAscan-SE (version 1.1) [41], and (3) alignment of clusters to Rfam sequences using HMMER (version 3.0) [42] and INFERNAL (version 1.0.2). The criteria for identification of known ncRNAs were as follows: (1) percentage of ORF less than 80%, (2) no hits in NR, (3) not mRNA, and (4) with homologues in Rfam [Eval (HMMER and INFERNAL) < 0.01]. For new ncRNA identification, the criteria were as follows: (1) percentage of ORF less than 80%, (2) no hits in NR, (3) not mRNA, (4) not rRNA, (5) not tRNA, and (6) no hits in Rfam (Eval > 0.01).

### Analysis of snRNAs folding and predication of snoRNAs putative targets

*T. rubrum* snRNAs are compared with the homologs in other fungi using the multiple sequence alignment software ClustalW2. The secondary structures of aligned sequences are predicted by RNAalifold [28]. The putative targets of snoRNAs were predicted by Snoscan and SnoGPS programs [17,18]. The potential target sequences as the 5.8S, 18S, and 25S rRNAs of *T. rubrum* were downloaded from GenBank under the accession number JX431933.

To predict the two classes of snoRNAs and their putative targets in our data, we used the Snoscan and SnoGPS programs, defining the potential target sequences as the 5.8S, 18S, and 25S rRNAs of *T. rubrum* and all snRNAs identified in our data [17,18].

### Northern blot analysis

For the northern blot analysis, 10 μg of total RNA was separated by electrophoresis on an 8% polyacrylamide gel containing 7 M urea and then electrotransferred onto a nylon membrane (Hybond-N+; Amersham) using a semi-dry blotting apparatus (BioRad). A total of 24–30 mer DNA oligonucleotides antisense to snRNAs and 15 randomly selected ncRNA candidates were end-labelled with (γ^32^P)-ATP and hybridised at 45°C for 16 hr. After stringency washes, the blots were exposed to phosphor storage screens, which were then scanned with a Typhoon 9200 imager (GE Healthcare).

### Nucleotide sequence accession numbers

The 352 ncRNAs sequences of *T. rubrum* were submitted to GenBank under the following accession numbers: KC352999 – KC353350.

## Competing interests

The authors declare that they have no competing interests.

## Authors’ contributions

TL, TX and XX performed experiments. XR, TX and JY analyzed and interpreted data. JD and LS carried out the ncRNA cDNA sequencing. TL and XR wrote the paper. RC and QJ proposed the research goal, supervised the whole studies and provided a critical review of the manuscript. All authors read and approved the final manuscript.

## Supplementary Material

Additional file 1: Table S1 Detailed information on ncRNAs identified in *T. rubrum.* Click here for file

Additional file 2: Figure S2 Secondary structure predictions of aligned snRNAs. Click here for file

Additional file 3: Figure S3 Potential base-paring between H/ACA box snoRNAs and rRNAs predicted by snoGPS. Click here for file

Additional file 4: Table S4 Conversed sncRNAs in all seven dermatophytes. Click here for file
