# Supplementary material for: Identification and characterisation of non-coding small RNAs in the pathogenic filamentous fungus Trichophyton rubrum
Source: BMC Genomics. 2013 Dec 30;14:931. doi: 10.1186/1471-2164-14-931 (PMC3890542; doi:10.1186/1471-2164-14-931)
Supplement: Additional file 1: Table S1 — Detailed information on ncRNAs identified in T. rubrum. [file 1471-2164-14-931-S1.pdf]

S1. Detail information of ncRNAs identified in *T.rubrum*

					Genome Location			Homology name in Rfam			
Name	Gene Bank Accession	Length	Abundance*	Reads	Chromosome	start	end	position	Accession	Homology Genes	Classes
Trnc_146	KC353007	222	Low	1	supercont2.1	707117	706896	3′UTR	no	no	ncRNA
Trnc_184	KC353011	142	Low	1	supercont2.1	887899	887758	5′ UTR	no	no	ncRNA
Trnc_190	KC353012	103	Low	1	supercont2.1	928714	928816	Intron	no	no	ncRNA
Trnc_311	KC353019	221	Low	1	supercont2.1	1541786	1541566	3′UTR	no	no	ncRNA
Trnc_500	KC353035	203	Low	1	supercont2.1	2298649	2298447	Intron	no	no	ncRNA
Trnc_602	KC353040	84	Low	1	supercont2.1	2678737	2678820	Intergenic	no	no	ncRNA
Trnc_611	KC353042	104	Low	1	supercont2.1	2710988	2711091	Intron	no	no	ncRNA
Trnc_691	KC353047	135	Low	1	supercont2.1	3142475	3142609	5′ UTR	no	no	ncRNA
Trnc_718	KC353048	122	Low	1	supercont2.1	3334692	3334571	Intron	no	no	ncRNA
Trnc_733	KC353049	174	Low	1	supercont2.1	3371115	3371288	3′UTR	no	no	ncRNA
Trnc_821	KC353055	210	Low	1	supercont2.1	3768831	3768622	Intergenic	no	no	ncRNA
Trnc_961	KC353065	177	Low	1	supercont2.10	578009	577833	5′UTR	no	no	ncRNA
Trnc_1098	KC353073	287	Low	1	supercont2.11	256290	256576	5′UTR	no	no	ncRNA
Trnc_1167	KC353076	177	Low	1	supercont2.11	544895	545071	3′UTR	no	no	ncRNA
Trnc_1211	KC353077	165	Low	1	supercont2.11	718257	718093	Intron	no	no	ncRNA
Trnc_1243	KC353080	133	Low	1	supercont2.12	145908	145776	Intron	no	no	ncRNA
Trnc_1249	KC353081	165	Low	1	supercont2.12	174997	174833	5′ UTR	no	no	ncRNA
Trnc_1293	KC353085	116	Low	1	supercont2.13	8434	8319	3′UTR	no	no	ncRNA
Trnc_1414	KC353096	68	Low	1	supercont2.16	81523	81590	3′ UTR	no	no	ncRNA
Trnc_1452	KC353102	84	Low	1	supercont2.18	17826	17909	5′UTR	no	no	ncRNA
Trnc_1456	KC353103	94	Low	1	supercont2.18	53193	53100	3′UTR	no	no	ncRNA
Trnc_1504	KC353107	193	Low	1	supercont2.2	231697	231889	3′UTR	no	no	ncRNA
Trnc_1509	KC353108	110	Low	1	supercont2.2	284655	284546	Intron	no	no	ncRNA
Trnc_1545	KC353109	192	Low	1	supercont2.2	458300	458109	5′ UTR	no	no	ncRNA
Trnc_1581	KC353113	190	Low	1	supercont2.2	672351	672540	3′UTR	no	no	ncRNA
Trnc_1634	KC353117	239	Low	1	supercont2.2	983863	983625	5′UTR	no	no	ncRNA
Trnc_1746	KC353126	250	Low	1	supercont2.2	1640240	1640489	5′UTR	no	no	ncRNA
Trnc_1811	KC353136	382	Low	1	supercont2.2	1888828	1889209	3′UTR	no	no	ncRNA
Trnc_2004	KC353146	129	Low	1	supercont2.3	63110	63238	3′UTR	no	no	ncRNA
Trnc_2058	KC353152	206	Low	1	supercont2.3	369951	369746	3′UTR	no	no	ncRNA
Trnc_2115	KC353155	253	Low	1	supercont2.3	581055	580803	5′ UTR	no	no	ncRNA
Trnc_2207	KC353162	89	Low	1	supercont2.3	1060294	1060206	5′ UTR	no	no	ncRNA
Trnc_2347	KC353168	176	Low	1	supercont2.3	1692947	1693122	5′ UTR	no	no	ncRNA
Trnc_2350	KC353169	132	Low	1	supercont2.3	1714306	1714175	5′ UTR	no	no	ncRNA
Trnc_2354	KC353170	152	Low	1	supercont2.3	1735194	1735043	Intron	no	no	ncRNA
Trnc_2366	KC353171	152	Low	1	supercont2.3	1811999	1811848	3′UTR	no	no	ncRNA
Trnc_2398	KC353174	106	Low	1	supercont2.3	1952087	1952192	Intron	no	no	ncRNA
Trnc_2431	KC353179	166	Low	1	supercont2.3	2070250	2070085	3′UTR	no	no	ncRNA
Trnc_2437	KC353180	160	Low	1	supercont2.3	2077721	2077562	3′UTR	no	no	ncRNA
Trnc_2448	KC353183	161	Low	1	supercont2.3	2123075	2122915	5′ UTR	no	no	ncRNA
Trnc_2501	KC353189	223	Low	1	supercont2.3	2469151	2468929	5′ UTR	no	no	ncRNA
Trnc_2683	KC353215	105	Low	1	supercont2.4	172556	172660	Intron	no	no	ncRNA
Trnc_2786	KC353224	152	Low	1	supercont2.4	709864	710015	Intergenic	no	no	ncRNA
Trnc_2821	KC353225	268	Low	1	supercont2.4	837487	837220	5′ UTR	no	no	ncRNA
Trnc_2890	KC353230	242	Low	1	supercont2.4	1157692	1157451	Intron	no	no	ncRNA
Trnc_2945	KC353236	166	Low	1	supercont2.4	1420896	1421061	5′UTR	no	no	ncRNA
Trnc_2952	KC353237	221	Low	1	supercont2.4	1490648	1490428	3′UTR	no	no	ncRNA
Trnc_3043	KC353243	162	Low	1	supercont2.4	1878388	1878227	3′UTR	no	no	ncRNA
Trnc_3096	KC353244	201	Low	1	supercont2.4	2153644	2153444	3′UTR	no	no	ncRNA
Trnc_3149	KC353249	193	Low	1	supercont2.5	305346	305154	5′UTR	no	no	ncRNA
Trnc_3157	KC353251	177	Low	1	supercont2.5	328773	328597	Intron	no	no	ncRNA
Trnc_3176	KC353252	137	Low	1	supercont2.5	423569	423705	3′UTR	no	no	ncRNA
Trnc_3178	KC353253	125	Low	1	supercont2.5	430082	430206	3′UTR	no	no	ncRNA
Trnc_3252	KC353259	228	Low	1	supercont2.5	787932	787705	3′UTR	no	no	ncRNA
Trnc_3256	KC353260	174	Low	1	supercont2.5	795747	795920	Intergenic	no	no	ncRNA
Trnc_3348	KC353263	226	Low	1	supercont2.5	1236562	1236337	5′UTR	no	no	ncRNA
Trnc_3392	KC353266	180	Low	1	supercont2.5	1543669	1543848	Intron	no	no	ncRNA
Trnc_3468	KC353271	186	Low	1	supercont2.6	313965	313780	3′UTR	no	no	ncRNA
Trnc_3484	KC353272	141	Low	1	supercont2.6	443757	443617	3′UTR	no	no	ncRNA
Trnc_3495	KC353273	230	Low	1	supercont2.6	522568	522797	5′UTR	no	no	ncRNA
Trnc_3579	KC353277	70	Low	1	supercont2.6	1008356	1008287	3′ UTR	no	no	ncRNA
Trnc_3637	KC353281	113	Low	1	supercont2.6	1368764	1368876	3′UTR	no	no	ncRNA
Trnc_3721	KC353289	155	Low	1	supercont2.7	426292	426446	3′UTR	no	no	ncRNA
Trnc_3728	KC353291	219	Low	1	supercont2.7	447410	447628	5′UTR	no	no	ncRNA
Trnc_3807	KC353297	143	Low	1	supercont2.7	1035866	1036008	5′UTR	no	no	ncRNA
Trnc_3815	KC353298	480	Low	1	supercont2.7	1070408	1069929	3′UTR	no	no	ncRNA
Trnc_3841	KC353300	209	Low	1	supercont2.7	1169120	1169328	3′UTR	no	no	ncRNA
Trnc_3851	KC353302	234	Low	1	supercont2.7	1244362	1244129	3′UTR	no	no	ncRNA
Trnc_4091	KC353321	145	Low	1	supercont2.8	1021753	1021609	3′UTR	no	no	ncRNA
Trnc_4120	KC353325	141	Low	1	supercont2.8	1178872	1178732	3′UTR	no	no	ncRNA
Trnc_4160	KC353327	117	Low	1	supercont2.9	169304	169420	3′UTR	no	no	ncRNA
Trnc_4165	KC353328	173	Low	1	supercont2.9	181512	181684	3′UTR	no	no	ncRNA
Trnc_4187	KC353332	223	Low	1	supercont2.9	331964	331742	3′UTR	no	no	ncRNA
Trnc_4219	KC353335	104	Low	1	supercont2.9	449429	449532	5′UTR	no	no	ncRNA
Trnc_4228	KC353336	123	Low	1	supercont2.9	533214	533336	5′UTR	no	no	ncRNA
Trnc_4239	KC353337	101	Low	1	supercont2.9	590614	590514	Intron	no	no	ncRNA
Trnc_4339	KC353350	140	Low	1	supercont2.9	1003602	1003463	3′UTR	no	no	ncRNA
Trnc_20	KC353000	94	Low	1	supercont2.1	48466	48559	5′UTR	no	no	ncRNA
Trnc_95	KC353005	242	Low	1	supercont2.1	436682	436923	3′UTR	no	no	ncRNA
Trnc_149	KC353008	205	Low	1	supercont2.1	709149	708945	5′ UTR	no	no	ncRNA
Trnc_268	KC353016	153	Low	1	supercont2.1	1349728	1349576	3′UTR	no	no	ncRNA
Trnc_369	KC353023	75	Low	1	supercont2.1	1744719	1744645	3′UTR	no	no	ncRNA
Trnc_440	KC353028	172	Low	1	supercont2.1	2014865	2014694	3′UTR	no	no	ncRNA
Trnc_789	KC353052	248	Low	1	supercont2.1	3618013	3618260	5′ UTR	no	no	ncRNA

Trnc_1032	KC353071	85	Low	1	supercont2.10	879574	879490	Intron	no	no	ncRNA
Trnc_1344	KC353087	289	Low	1	supercont2.13	260355	260643	3′UTR	no	no	ncRNA
Trnc_1463	KC353104	99	Low	1	supercont2.2	54765	54863	3′UTR	no	no	ncRNA
Trnc_1490	KC353106	128	Low	1	supercont2.2	178065	178192	3′UTR	no	no	ncRNA
Trnc_1562	KC353112	254	Low	1	supercont2.2	553962	553709	3′UTR	no	no	ncRNA
Trnc_1639	KC353118	107	Low	1	supercont2.2	1019985	1019879	Intergenic	no	no	ncRNA
Trnc_1661	KC353119	169	Low	1	supercont2.2	1163041	1162873	Intron	no	no	ncRNA
Trnc_1736	KC353125	115	Low	1	supercont2.2	1574471	1574357	5′UTR	no	no	ncRNA
Trnc_1792	KC353132	251	Low	1	supercont2.2	1856556	1856806	Intron	no	no	ncRNA
Trnc_1793	KC353133	136	Low	1	supercont2.2	1857072	1857207	5′ UTR	no	no	ncRNA
Trnc_2100	KC353154	134	Low	1	supercont2.3	492158	492291	Intron	no	no	ncRNA
Trnc_2244	KC353163	93	Low	1	supercont2.3	1220514	1220606	3′UTR	no	no	ncRNA
Trnc_2489	KC353187	317	Low	1	supercont2.3	2384439	2384755	3′UTR	no	no	ncRNA
Trnc_2603	KC353201	137	Low	1	supercont2.35	73	209	3′UTR	no	no	ncRNA
Trnc_2626	KC353208	135	Low	1	supercont2.36	11611	11745	Intergenic	no	no	ncRNA
Trnc_2678	KC353214	172	Low	1	supercont2.4	127188	127017	3′UTR	no	no	ncRNA
Trnc_2704	KC353218	80	Low	1	supercont2.4	350218	350297	Intron	no	no	ncRNA
Trnc_2976	KC353239	132	Low	1	supercont2.4	1594325	1594194	3′UTR	no	no	ncRNA
Trnc_3100	KC353245	172	Low	1	supercont2.5	36493	36322	5′ UTR	no	no	ncRNA
Trnc_3101	KC353246	136	Low	1	supercont2.5	52331	52466	5′UTR	no	no	ncRNA
Trnc_3690	KC353288	86	Low	1	supercont2.7	222180	222095	5′ UTR	no	no	ncRNA
Trnc_3795	KC353296	140	Low	1	supercont2.7	899924	899785	3′UTR	no	no	ncRNA
Trnc_3852	KC353303	92	Low	1	supercont2.7	1266049	1265958	5′ UTR	no	no	ncRNA
Trnc_3923	KC353309	101	Low	1	supercont2.8	272917	272817	Intron	no	no	ncRNA
Trnc_3984	KC353312	92	Low	1	supercont2.8	614427	614518	3′UTR	no	no	ncRNA
Trnc_3993	KC353313	94	Low	1	supercont2.8	638650	638743	3′UTR	no	no	ncRNA
Trnc_4052	KC353318	95	Low	1	supercont2.8	869939	870033	5′ UTR	no	no	ncRNA
Trnc_4214	KC353334	141	Low	1	supercont2.9	424473	424333	5′UTR	no	no	ncRNA
Trnc_2966	KC353238	103	Low	1	supercont2.4	1562900	1562798	3′UTR	no	no	ncRNA
Trnc_54	KC353001	97	Low	1	supercont2.1	188653	188749	3′UTR	no	no	ncRNA
Trnc_2504	KC353190	75	Low	1	supercont2.3	2479716	2479790	3′UTR	RF00461	IRES_VEGF_A	ncRNA; Cis-reg
Trnc_3129	KC353247	103	Low	1	supercont2.5	190440	190542	5′UTR	RF00465	JEV_hairpin	ncRNA; Cis-reg
Trnc_2565	KC353196	102	Low	1	supercont2.3	2757753	2757652	Intron	RF01068	mini-ykkC	ncRNA; Cis-reg
Trnc_2913	KC353232	82	Low	1	supercont2.4	1303343	1303262	Intron	RF01102	PK1-TEV_CVMV	ncRNA; Cis-reg
Trnc_4067	KC353320	86	Low	1	supercont2.8	927922	927837	5′UTR	RF00043	Plasmid_R1162	ncRNA; Cis-reg
Trnc_3269	KC353261	121	Low	1	supercont2.5	832731	832611	3′UTR	RF00180	REN-SRE	ncRNA; Cis-reg
Trnc_682	KC353045	99	Low	1	supercont2.1	3064174	3064076	3′UTR	RF00506	Thr_leader	ncRNA; Cis-reg
Trnc_85	KC353004	110	Low	1	supercont2.1	366825	366934	5′ UTR	RF00506	Thr_leader	ncRNA; Cis-reg
Trnc_1611	KC353116	133	Low	1	supercont2.2	859642	859774	5′ UTR	RF01077	TLS-PK2	ncRNA; Cis-reg
Trnc_3725	KC353290	139	Low	1	supercont2.7	440095	439957	3′UTR	RF01088	TLS-PK5	ncRNA; Cis-reg
Trnc_155	KC353009	102	Low	1	supercont2.1	749414	749313	Intron	RF00063	SscA	ncRNA; SscA RNA
Trnc_1881	KC353139	103	Low	1	supercont2.2	2312593	2312695	Intergenic	RF00953	mir-1497	ncRNA;pri- or pre-miRNA
Trnc_83	KC353003	94	Low	1	supercont2.1	365858	365951	5′ UTR	RF00660	mir-214	ncRNA;pri- or pre-miRNA
Trnc_465	KC353029	131	Low	1	supercont2.1	2097724	2097594	5′UTR	RF00660	mir-214	ncRNA;pri- or pre-miRNA
Trnc_3156	KC353250	91	Low	1	supercont2.5	323403	323313	3′UTR	RF00660	mir-214	ncRNA;pri- or pre-miRNA
Trnc_2302	KC353166	93	Low	1	supercont2.3	1361651	1361743	Intron	RF00795	mir-43	ncRNA;pri- or pre-miRNA
Trnc_739	KC353050	158	Low	1	supercont2.1	3402590	3402433	5′UTR	RF00746	mir-454	ncRNA;pri- or pre-miRNA
Trnc_953	KC353062	109	Low	1	supercont2.10	557706	557598	Intergenic	RF00889	MIR533	ncRNA;pri- or pre-miRNA
Trnc_957	KC353064	85	Low	1	supercont2.10	559450	559366	Intron	RF00889	MIR533	ncRNA;pri- or pre-miRNA
Trnc_1006	KC353068	89	Low	1	supercont2.10	740838	740750	5′UTR	RF00889	MIR533	ncRNA;pri- or pre-miRNA
Trnc_3140	KC353248	84	Low	1	supercont2.5	259173	259090	5′ UTR	RF00889	MIR533	ncRNA;pri- or pre-miRNA
Trnc_3449	KC353270	85	Low	1	supercont2.6	164860	164776	5′ UTR	RF00889	MIR533	ncRNA;pri- or pre-miRNA
Trnc_4178	KC353329	99	Low	1	supercont2.9	276818	276916	Intron	RF00889	MIR533	ncRNA;pri- or pre-miRNA
Trnc_385	KC353024	98	Low	1	supercont2.1	1818863	1818960	3′UTR	RF00990	mir-552	ncRNA;pri- or pre-miRNA
Trnc_3783	KC353295	80	Low	1	supercont2.7	784466	784545	3′UTR	RF00990	mir-552	ncRNA;pri- or pre-miRNA
Trnc_161	KC353010	91	Low	1	supercont2.1	792780	792870	5′ UTR	no	mir-574	ncRNA;pri- or pre-miRNA
Trnc_252	KC353015	158	Low	1	supercont2.1	1248092	1247935	Intron	RF01059	mir-598	ncRNA;pri- or pre-miRNA
Trnc_492	KC353033	98	Low	1	supercont2.1	2238242	2238145	Intron	RF01059	mir-598	ncRNA;pri- or pre-miRNA
Trnc_512	KC353036	195	Low	1	supercont2.1	2323310	2323504	Intron	no	mir-598	ncRNA;pri- or pre-miRNA
Trnc_2862	KC353229	70	Low	1	supercont2.4	1094194	1094263	Intron	RF01059	mir-598	ncRNA;pri- or pre-miRNA
Trnc_3569	KC353275	297	Low	1	supercont2.6	934438	934142	Intron	RF01059	mir-598	ncRNA;pri- or pre-miRNA
Trnc_4208	KC353333	179	Low	1	supercont2.9	416906	416728	Intergenic	RF01059	mir-598	ncRNA;pri- or pre-miRNA
Trnc_1561	KC353111	120	Low	1	supercont2.2	547418	547537	3′UTR	RF01059	mir-598	ncRNA;pri- or pre-miRNA
Trnc_1401	KC353093	88	Low	1	supercont2.15	158007	158094	3′UTR	RF00844	mir-67	ncRNA;pri- or pre-miRNA
Trnc_478	KC353031	92	Low	1	supercont2.1	2144845	2144936	5′UTR	no	no	ncRNA
Trnc_3613	KC353279	134	Low	1	supercont2.6	1217129	1216996	5′UTR	no	no	no
Trnc_3614	KC353280	80	Low	1	supercont2.6	1218983	1218904	Intron	no	no	no
Trnc_3943	KC353310	94	Low	1	supercont2.8	378650	378557	Intron	no	no	no
Trnc_3959	KC353311	82	Low	1	supercont2.8	495724	495805	3′UTR	no	no	no
Trnc_4002	KC353315	158	Low	1	supercont2.8	682342	682499	Intron	no	no	no
Trnc_1357	KC353089	208	Low	1	supercont2.14	153790	153997	5′UTR	RF00621	CoTC_ribozyme	ribozyme
Trnc_313	KC353020	77	Low	1	supercont2.1	1543338	1543262	5′ UTR	RF01118	PK-G12rRNA	rRNA
Trnc_2018	KC353149	306	Low	1	supercont2.3	117035	116730	3′UTR	no	no	snoRNA;CD-box
Trnc_2179	KC353160	431	Low	1	supercont2.3	961995	961565	Intergenic	no	no	snoRNA;CD-box
Trnc_2283	KC353165	233	Low	1	supercont2.3	1301587	1301819	5′ UTR	no	no	snoRNA;CD-box
Trnc_2405	KC353175	317	Low	1	supercont2.3	1975149	1975465	3′UTR	no	no	snoRNA;CD-box
Trnc_2691	KC353216	158	Low	1	supercont2.4	233433	233276	Intergenic	no	no	snoRNA;CD-box
Trnc_4250	KC353339	192	Low	1	supercont2.9	658585	658394	3′UTR	no	no	snoRNA;CD-box
Trnc_2782	KC353223	128	Low	1	supercont2.4	669565	669438	5′ UTR	RF00630	P26	snoRNA;CD-box
Trnc_608	KC353041	234	Low	1	supercont2.1	2701229	2701462	3′UTR	RF01202	sn2991	snoRNA;CD-box
Trnc_3667	KC353285	191	Low	1	supercont2.7	59063	59253	3′UTR	RF00529	noMe28S-Am258	snoRNA;CD-box
Trnc_1603	KC353115	358	Low	1	supercont2.2	766347	766704	3′UTR	RF00345	snoR1	snoRNA;CD-box
Trnc_1709	KC353124	154	Low	1	supercont2.2	1400380	1400533	5′ UTR	RF01193	snoR20a	snoRNA;CD-box
Trnc_2027	KC353150	96	Low	1	supercont2.3	166668	166763	Intron	RF01281	snoR35	snoRNA;CD-box
Trnc_1449	KC353101	234	Low	1	supercont2.17	97081	97314	5′UTR	RF01191	SNORD121A	snoRNA;CD-box
Trnc_3227	KC353256	139	Low	1	supercont2.5	625518	625380	Intron	RF00594	SNORD86	snoRNA;CD-box
Trnc_1299	KC353086	109	Low	1	supercont2.13	24837	24729	Intron	RF00593	snoU83B	snoRNA;CD-box
Trnc_3438	KC353269	173	Low	1	supercont2.6	91295	91467	5′UTR	RF01291	snoU97	snoRNA;CD-box

Trnc_2011	KC353147	127	Low	1	supercont2.3	74633	74759	3′UTR	RF00441	snoZ242	snoRNA;CD-box
Trnc_4316	KC353347	97	Low	1	supercont2.9	861468	861372	5′UTR	RF01223	snR13	snoRNA;CD-box
Trnc_1366	KC353091	215	Low	1	supercont2.14	179253	179467	3′UTR	RF01152	sR1	snoRNA;CD-box
Trnc_1841	KC353138	143	Low	1	supercont2.2	2090171	2090313	3′UTR	RF01144	sR17	snoRNA;CD-box
Trnc_1560	KC353110	77	Low	1	supercont2.2	546818	546894	3′UTR	RF01139	sR2	snoRNA;CD-box
Trnc_3654	KC353284	191	Low	1	supercont2.7	14823	15013	3′UTR	RF01140	sR20	snoRNA;CD-box
Trnc_3833	KC353299	109	Low	1	supercont2.7	1124537	1124429	3′UTR	RF01273	sR34	snoRNA;CD-box
Trnc_415	KC353027	103	Low	1	supercont2.1	1918108	1918210	Intron	RF01121	Sr38	snoRNA;CD-box
Trnc_5	KC352999	289	Low	1	supercont2.1	19982	20270	5′ UTR	no	no	snoRNA;HACA-box
Trnc_64	KC353002	306	Low	1	supercont2.1	267027	267332	3′UTR	no	no	snoRNA;HACA-box
Trnc_203	KC353013	308	Low	1	supercont2.1	996485	996178	5′ UTR	no	no	snoRNA;HACA-box
Trnc_910	KC353060	468	Low	1	supercont2.10	343107	343574	5′UTR	no	no	snoRNA;HACA-box
Trnc_920	KC353061	310	Low	1	supercont2.10	389299	389608	3′UTR	no	no	snoRNA;HACA-box
Trnc_1355	KC353088	371	Low	1	supercont2.14	142837	142467	5′UTR	no	no	snoRNA;HACA-box
Trnc_2045	KC353151	228	Low	1	supercont2.3	296293	296520	5′ UTR	no	no	snoRNA;HACA-box
Trnc_2452	KC353184	323	Low	1	supercont2.3	2170039	2169717	3′UTR	no	no	snoRNA;HACA-box
Trnc_2579	KC353198	349	Low	1	supercont2.3	2792710	2793058	5′UTR	no	no	snoRNA;HACA-box
Trnc_2898	KC353231	393	Low	1	supercont2.4	1199167	1198775	Intron	no	no	snoRNA;HACA-box
Trnc_3005	KC353241	214	Low	1	supercont2.4	1748930	1749143	5′UTR	no	no	snoRNA;HACA-box
Trnc_3218	KC353255	332	Low	1	supercont2.5	584674	585005	5′UTR	no	no	snoRNA;HACA-box
Trnc_3387	KC353265	226	Low	1	supercont2.5	1472165	1472390	3′UTR	no	no	snoRNA;HACA-box
Trnc_3509	KC353274	433	Low	1	supercont2.6	608530	608098	5′UTR	no	no	snoRNA;HACA-box
Trnc_3585	KC353278	281	Low	1	supercont2.6	1065274	1064994	Intron	no	no	snoRNA;HACA-box
Trnc_1407	KC353095	234	Low	1	supercont2.16	54707	54474	3′UTR	no	no	snoRNA;HACA-box
Trnc_2531	KC353194	75	Low	1	supercont2.3	2617075	2617001	Intron	no	no	snoRNA;HACA-box
Trnc_2596	KC353200	324	Low	1	supercont2.3	2882125	2881802	3′UTR	no	no	snoRNA;HACA-box
Trnc_2636	KC353210	203	Low	1	supercont2.36	19276	19478	Intergenic	no	no	snoRNA;HACA-box
Trnc_2443	KC353182	106	Low	1	supercont2.3	2090244	2090349	Intron	RF00428	SNORA38	snoRNA;HACA-box
Trnc_2172	KC353159	126	Low	1	supercont2.3	922150	922025	Intron	RF00406	SNORA42	snoRNA;HACA-box
Trnc_1370	KC353092	133	Low	1	supercont2.14	187697	187565	5′UTR	RF01134	sR30	snoRNA;HACA-box
Trnc_568	KC353038	128	Low	1	supercont2.1	2533804	2533677	3′UTR	RF00565	SCARNA3	snoRNA;scaRNA
Trnc_411	KC353026	193	Low	2	supercont2.1	1899519	1899327	Intergenic	no	no	ncRNA
Trnc_872	KC353056	239	Low	2	supercont2.10	23790	23552	5′ UTR	no	no	ncRNA
Trnc_873	KC353057	164	Low	2	supercont2.10	48543	48706	5′UTR	no	no	ncRNA
Trnc_877	KC353058	97	Low	2	supercont2.10	76955	77051	3′UTR	no	no	ncRNA
Trnc_956	KC353063	241	Low	2	supercont2.10	559285	559525	5′UTR	no	no	ncRNA
Trnc_997	KC353067	216	Low	2	supercont2.10	732727	732512	5′ UTR	no	no	ncRNA
Trnc_1114	KC353074	104	Low	2	supercont2.11	328144	328247	3′UTR	no	no	ncRNA
Trnc_1415	KC353097	140	Low	2	supercont2.16	82202	82063	5′ UTR	no	no	ncRNA
Trnc_1426	KC353099	193	Low	2	supercont2.16	111833	111641	3′UTR	no	no	ncRNA
Trnc_1702	KC353123	375	Low	2	supercont2.2	1373166	1372792	3′UTR	no	no	ncRNA
Trnc_1801	KC353134	209	Low	2	supercont2.2	1873103	1873311	Intron	no	no	ncRNA
Trnc_1939	KC353143	203	Low	2	supercont2.2	2642605	2642403	Intergenic	no	no	ncRNA
Trnc_1953	KC353144	348	Low	2	supercont2.2	2715124	2714777	5′ UTR	no	no	ncRNA
Trnc_2014	KC353148	168	Low	2	supercont2.3	75171	75338	3′UTR	no	no	ncRNA
Trnc_2186	KC353161	84	Low	2	supercont2.3	986525	986442	Intron	no	no	ncRNA
Trnc_2397	KC353173	296	Low	2	supercont2.3	1950899	1950604	Intron	no	no	ncRNA
Trnc_2417	KC353176	215	Low	2	supercont2.3	2020355	2020141	Intron	no	no	ncRNA
Trnc_2463	KC353185	264	Low	2	supercont2.3	2177418	2177155	5′UTR	no	no	ncRNA
Trnc_2471	KC353186	157	Low	2	supercont2.3	2234172	2234016	Intron	no	no	ncRNA
Trnc_2676	KC353213	156	Low	2	supercont2.4	110438	110593	3′UTR	no	no	ncRNA
Trnc_2721	KC353219	258	Low	2	supercont2.4	476938	476681	3′UTR	no	no	ncRNA
Trnc_3643	KC353282	109	Low	2	supercont2.6	1430407	1430299	3′UTR	no	no	ncRNA
Trnc_3648	KC353283	122	Low	2	supercont2.6	1497645	1497766	3′UTR	no	no	ncRNA
Trnc_3681	KC353287	188	Low	2	supercont2.7	185159	185346	3′UTR	no	no	ncRNA
Trnc_3909	KC353307	355	Low	2	supercont2.8	190791	191145	5′ UTR	no	no	ncRNA
Trnc_4066	KC353319	230	Low	2	supercont2.8	925945	926174	3′UTR	no	no	ncRNA
Trnc_4093	KC353322	141	Low	2	supercont2.8	1041751	1041891	3′UTR	no	no	ncRNA
Trnc_4106	KC353323	126	Low	2	supercont2.8	1131640	1131765	5′ UTR	no	no	ncRNA
Trnc_4247	KC353338	193	Low	2	supercont2.9	612229	612421	5′ UTR	no	no	ncRNA
Trnc_401	KC353025	225	Low	2	supercont2.1	1846758	1846534	3′UTR	no	no	ncRNA
Trnc_589	KC353039	284	Low	2	supercont2.1	2667010	2666727	3′UTR	no	no	ncRNA
Trnc_1213	KC353078	327	Low	2	supercont2.11	755714	755388	3′UTR	no	no	ncRNA
Trnc_1231	KC353079	156	Low	2	supercont2.12	110235	110390	3′UTR	no	no	ncRNA
Trnc_1403	KC353094	134	Low	2	supercont2.16	20810	20677	3′UTR	no	no	ncRNA
Trnc_1772	KC353128	202	Low	2	supercont2.2	1787764	1787563	3′UTR	no	no	ncRNA
Trnc_1808	KC353135	283	Low	2	supercont2.2	1884802	1885084	Intron	no	no	ncRNA
Trnc_1887	KC353140	184	Low	2	supercont2.2	2365061	2364878	5′UTR	no	no	ncRNA
Trnc_2136	KC353157	159	Low	2	supercont2.3	717783	717941	5′ UTR	no	no	ncRNA
Trnc_2376	KC353172	123	Low	2	supercont2.3	1846538	1846660	5′ UTR	no	no	ncRNA
Trnc_2522	KC353191	106	Low	2	supercont2.3	2580763	2580658	3′UTR	no	no	ncRNA
Trnc_2649	KC353212	79	Low	2	supercont2.36	23976	24054	Intergenic	no	no	ncRNA
Trnc_2778	KC353222	126	Low	2	supercont2.4	658938	658813	5′UTR	no	no	ncRNA
Trnc_3782	KC353294	96	Low	2	supercont2.7	784365	784460	3′UTR	no	no	ncRNA
Trnc_1777	KC353130	206	Low	2	supercont2.2	1789893	1789688	3′UTR	RF00458	IRES_Cripavirus	ncRNA; Cis-reg
Trnc_206	KC353014	109	Low	2	supercont2.1	1002398	1002506	Intron	RF00889	MIR533	ncRNA;pri- or pre-miRNA
Trnc_2918	KC353233	160	Low	2	supercont2.4	1319018	1319177	5′UTR	RF00889	MIR533	ncRNA;pri- or pre-miRNA
Trnc_112	KC353006	100	Low	2	supercont2.1	553233	553134	3′UTR	RF01059	mir-598	ncRNA;pri- or pre-miRNA
Trnc_685	KC353046	100	Low	2	supercont2.1	3092534	3092633	Intron	RF01059	mir-598	ncRNA;pri- or pre-miRNA
Trnc_1983	KC353145	86	Low	2	supercont2.2	2887537	2887452	3′UTR	RF01059	mir-598	ncRNA;pri- or pre-miRNA
Trnc_2700	KC353217	128	Low	2	supercont2.4	311242	311115	Intron	RF01010	mir-632	ncRNA;pri- or pre-miRNA
Trnc_3844	KC353301	102	Low	2	supercont2.7	1201652	1201551	Intron	no	no	no
Trnc_2569	KC353197	192	Low	2	supercont2.3	2759920	2759729	Intron	no	no	snoRNA;CD-box
Trnc_2498	KC353188	172	Low	2	supercont2.3	2451919	2452090	5′UTR	RF00527	snoMe28S-G3255	snoRNA;CD-box
Trnc_985	KC353066	153	Low	2	supercont2.10	686423	686575	Intron	RF00494	snoU2_19	snoRNA;CD-box
Trnc_640	KC353043	129	Low	2	supercont2.1	2869815	2869687	3′UTR	RF00300	snoZ221	snoRNA;CD-box
Trnc_1272	KC353084	265	Low	2	supercont2.12	280712	280448	Intron	RF01152	sR1	snoRNA;CD-box
Trnc_2594	KC353199	143	Low	2	supercont2.3	2859175	2859033	Intron	RF01305	sR51	snoRNA;CD-box

Trnc_2606	KC353202	280	Low	2	supercont2.36	2106	2385	Intergenic	no	no	snoRNA;HACA-box
Trnc_3023	KC353242	182	Low	2	supercont2.4	1839416	1839597	3′UTR	no	no	snoRNA;HACA-box
Trnc_2640	KC353211	71	Low	2	supercont2.36	21309	21379	Intergenic	RF00005	tRNA	tRNA
Trnc_1423	KC353098	305	Low	3	supercont2.16	86158	86462	3′UTR	no	no	ncRNA
Trnc_2126	KC353156	236	Low	3	supercont2.3	651208	650973	Intron	no	no	ncRNA
Trnc_2757	KC353220	89	Low	3	supercont2.4	610530	610618	3′UTR	no	no	ncRNA
Trnc_477	KC353030	151	Low	3	supercont2.1	2139613	2139463	3′UTR	no	no	ncRNA
Trnc_2438	KC353181	460	Low	3	supercont2.3	2078096	2078555	5′ UTR	no	no	ncRNA
Trnc_2776	KC353221	186	Low	3	supercont2.4	655157	655342	5′UTR	no	no	ncRNA
Trnc_3228	KC353257	204	Low	3	supercont2.5	659623	659420	3′UTR	no	no	ncRNA
Trnc_4338	KC353349	135	Low	3	supercont2.9	997085	996951	5′UTR	RF01059	mir-598	ncRNA;pri- or pre-miRNA
Trnc_3911	KC353308	80	Low	3	supercont2.8	194694	194773	Intron	RF00213	snoR38	snoRNA;CD-box
Trnc_3855	KC353305	288	Low	3	supercont2.7	1281447	1281734	Intron	RF01127	sR42	snoRNA;CD-box
Trnc_1698	KC353122	360	Low	3	supercont2.2	1345609	1345968	Intron	no	no	snoRNA;HACA-box
Trnc_488	KC353032	95	Low	4	supercont2.1	2219199	2219293	3′UTR	no	no	ncRNA
Trnc_553	KC353037	200	Low	4	supercont2.1	2462913	2462714	5′ UTR	no	no	ncRNA
Trnc_1771	KC353127	219	Low	4	supercont2.2	1787241	1787023	3′UTR	no	no	ncRNA
Trnc_2609	KC353203	255	Low	4	supercont2.36	4048	4302	Intergenic	no	no	ncRNA
Trnc_3382	KC353264	286	Low	4	supercont2.5	1463205	1462920	Intron	no	no	ncRNA
Trnc_4150	KC353326	398	Low	4	supercont2.9	166263	165866	5′ UTR	no	no	ncRNA
Trnc_1688	KC353121	223	Low	4	supercont2.2	1314492	1314270	3′UTR	no	no	ncRNA
Trnc_1888	KC353141	290	Low	4	supercont2.2	2381176	2381465	3′UTR	no	no	ncRNA
Trnc_2523	KC353192	270	Low	4	supercont2.3	2581274	2581005	Intron	RF00684	mir-122	ncRNA;pri- or pre-miRNA
Trnc_2163	KC353158	137	Low	4	supercont2.3	874758	874894	Intron	RF00647	MIR164	ncRNA;pri- or pre-miRNA
Trnc_1089	KC353072	145	Low	4	supercont2.11	218126	217982	Intron	RF01059	mir-598	ncRNA;pri- or pre-miRNA
Trnc_2999	KC353240	290	Low	4	supercont2.4	1720998	1721287	5′UTR	no	no	snoRNA;HACA-box
Trnc_4007	KC353317	239	Low	4	supercont2.8	722404	722166	3′UTR	no	no	snoRNA;HACA-box
Trnc_3999	KC353314	178	Low	5	supercont2.8	672734	672557	3′UTR	no	no	ncRNA
Trnc_1271	KC353083	242	Low	5	supercont2.12	280437	280196	Intron	RF01152	sR1	snoRNA;CD-box
Trnc_2618	KC353205	322	Low	5	supercont2.36	8062	8383	Intergenic	no	no	snoRNA;HACA-box
Trnc_2075	KC353153	96	Low	5	supercont2.3	425677	425772	Intron	RF00405	SNORA44	snoRNA;HACA-box
Trnc_2309	KC353167	94	Low	6	supercont2.3	1380352	1380445	5′UTR	no	no	ncRNA
Trnc_2935	KC353234	162	Low	9	supercont2.4	1403494	1403333	Intron	RF00220	Rhino_CRE	ncRNA; Cis-reg
Trnc_1776	KC353129	326	Low	9	supercont2.2	1789188	1788863	3′UTR	RF01231	snoR74	snoRNA;HACA-box
Trnc_4267	KC353346	100	Low	10	supercont2.9	703004	703103	3′UTR	no	no	snoRNA;CD-box
Trnc_3297	KC353262	138		12	supercont2.5	896392	896529	3′UTR	RF00610	SNORD110	snoRNA;CD-box
Trnc_2936	KC353235	246		16	supercont2.4	1403883	1403638	Intron	RF00312	snoZ206	snoRNA;CD-box
Trnc_1596	KC353114	611		18	supercont2.2	739588	740198	3′UTR	no	no	ncRNA
Trnc_494	KC353034	170		20	supercont2.1	2239950	2240119	5′ UTR	RF01059	mir-598	ncRNA;pri- or pre-miRNA
Trnc_1893	KC353142	344		21	supercont2.2	2393882	2393539	3′UTR	no	no	snoRNA;HACA-box
Trnc_2419	KC353177	204		22	supercont2.3	2045771	2045974	5′UTR	RF01125	sR4	snoRNA;CD-box
Trnc_4113	KC353324	681		22	supercont2.8	1152047	1152727	Intron	RF01274	sR45	snoRNA;CD-box
Trnc_2614	KC353204	285		23	supercont2.36	5177	5461	Intergenic	no	no	ncRNA
Trnc_4264	KC353345	88		23	supercont2.9	696179	696266	5′UTR	RF01207	snR73	snoRNA;CD-box
Trnc_892	KC353059	182		23	supercont2.10	182361	182542	3′UTR	RF00005	tRNA	tRNA
Trnc_2545	KC353195	119		27	supercont2.3	2657688	2657806	3′UTR	RF01188	snR56	snoRNA;CD-box
Trnc_4006	KC353316	251		28	supercont2.8	710950	711200	Intron	RF01263	snR191	snoRNA;HACA-box
Trnc_3778	KC353293	101		31	supercont2.7	777627	777727	5′ UTR	RF00471	snosnR48	snoRNA;CD-box
Trnc_3179	KC353254	207		39	supercont2.5	431925	431719	3′UTR	no	no	ncRNA
Trnc_315	KC353021	740		40	supercont2.1	1544520	1545259	3′UTR	no	no	ncRNA
Trnc_1008	KC353069	419		41	supercont2.10	748640	749058	3′UTR	RF00030	RNase_MRP	ribozyme
Trnc_3741	KC353292	180		42	supercont2.7	491853	492032	3′UTR	RF01247	snR32	snoRNA;HACA-box
Trnc_1157	KC353075	95		43	supercont2.11	539262	539356	Intron	RF00093	SNORD18	snoRNA;CD-box
Trnc_338	KC353022	135		44	supercont2.1	1643180	1643314	Intron	RF01223	snR13	snoRNA;CD-box
Trnc_2843	KC353227	225		56	supercont2.4	976176	976400	3′UTR	RF01251	snR3	snoRNA;HACA-box
Trnc_1010	KC353070	87		80	supercont2.10	749220	749306	3′UTR	RF00477	snosnR66	snoRNA;CD-box
Trnc_3425	KC353267	202		94	supercont2.6	22581	22782	3′UTR	no	no	snoRNA;CD-box
Trnc_2621	KC353206	406		97	supercont2.36	8934	9339	Intergenic	no	no	snoRNA;HACA-box
Trnc_1252	KC353082	181	High	104	supercont2.12	182834	182654	3′UTR	no	no	ncRNA
Trnc_1686	KC353120	133	High	126	supercont2.2	1303987	1304119	3′UTR	no	no	ncRNA
Trnc_4179	KC353330	657	High	129	supercont2.9	277309	276653	Intron	no	no	ncRNA
Trnc_4180	KC353331	467	High	129	supercont2.9	277728	277262	Intron	no	no	ncRNA
Trnc_4336	KC353348	162	High	137	supercont2.9	996654	996493	Intron	no	no	snoRNA;CD-box
Trnc_3573	KC353276	95	High	147	supercont2.6	964586	964680	Intron	RF00530	noMe28S-Cm264	snoRNA;CD-box
Trnc_1437	KC353100	264	High	153	supercont2.17	13253	15593	Intergenic	RF00015	U4	snRNA;splicing
Trnc_4260	KC353341	95	High	163	supercont2.9	695194	695288	Intergenic	RF01178	snoR77Y	snoRNA;CD-box
Trnc_774	KC353051	201	High	163	supercont2.1	3545014	3545214	3′UTR	RF00004	U2	snRNA;splicing
Trnc_1472	KC353105	188	High	170	supercont2.2	69663	69850	3′UTR	RF01258	snR10	snoRNA;HACA-box
Trnc_2265	KC353164	87	High	187	supercont2.3	1276133	1276219	Intron	RF01197	snR39	snoRNA;CD-box
Trnc_1825	KC353137	309	High	200	supercont2.2	1958330	1958022	3′UTR	no	no	snoRNA;CD-box
Trnc_2844	KC353228	239	High	225	supercont2.4	981025	981263	5′ UTR	no	no	ncRNA
Trnc_3677	KC353286	152	High	225	supercont2.7	144593	144744	Intron	no	no	ncRNA
Trnc_2622	KC353207	87	High	258	supercont2.36	10861	10947	Intergenic	RF00005	tRNA	tRNA
Trnc_2526	KC353193	321	High	288	supercont2.3	2582456	2582136	3′UTR	no	no	ncRNA
Trnc_1359	KC353090	97	High	290	supercont2.14	159345	159441	Intron	RF00475	snosnR69	snoRNA;CD-box
Trnc_2633	KC353209	597	High	297	supercont2.36	17132	17728	Intergenic	no	no	ncRNA
Trnc_817	KC353054	188	High	323	supercont2.1	3719705	3719892	3′UTR	no	no	snoRNA;HACA-box
Trnc_4263	KC353344	157	High	486	supercont2.9	695917	696073	Intergenic	RF00086	SNORD27	snoRNA;CD-box
Trnc_305	KC353018	97	High	579	supercont2.1	1515685	1515781	Intron	no	no	ncRNA
Trnc_4262	KC353343	273	High	603	supercont2.9	695588	695860	Intergenic	RF01185	snR75	snoRNA;CD-box
Trnc_3237	KC353258	164	High	673	supercont2.5	722162	721999	Intron	no	no	ncRNA
Trnc_3426	KC353268	98	High	761	supercont2.6	23000	23097	3′UTR	no	no	snoRNA;CD-box
Trnc_4259	KC353340	104	High	892	supercont2.9	693331	693434	5′ UTR	RF00276	SNORD52	snoRNA;CD-box
Trnc_293	KC353017	116	High	917	supercont2.1	1477742	1477627	3′UTR	no	no	ncRNA
Trnc_801	KC353053	488	High	919	supercont2.1	3681448	3681935	3′UTR	RF00012	U3	snoRNA;CD-box
Trnc_3853	KC353304	192	High	944	supercont2.7	1268084	1268275	3′UTR	no	no	ncRNA
Trnc_2421	KC353178	182	High	1060	supercont2.3	2046135	2046316	5′ UTR	RF00016	SNORD14	snoRNA;CD-box
Trnc_3904	KC353306	196	High	1238	supercont2.8	159538	159733	5′UTR	RF00003	U1	snRNA;splicing
Trnc_4261	KC353342	138	High	5548	supercont2.9	695445	695582	Intergenic	RF01209	snR76	snoRNA;CD-box

Trnc_1782	KC353131	104	High	9034	supercont2.2	1801544	1801647	3′UTR	RF00026	U6	snRNA;splicing
Trnc_2839	KC353226	186	High	11647	supercont2.4	948628	948443	5′ UTR	no	no	ncRNA
Trnc_681	KC353044	211	High	15583	supercont2.1	3061687	3061897	5′ UTR	RF00020	U5	snRNA;splicing

*Low: Reads number < 10, Low abundant ncRNAs; High: Reads number >100, High abundant ncRNAs
