# Supplementary material for: Identification and characterisation of non-coding small RNAs in the pathogenic filamentous fungus Trichophyton rubrum
Source: BMC Genomics. 2013 Dec 30;14:931. doi: 10.1186/1471-2164-14-931 (PMC3890542; doi:10.1186/1471-2164-14-931)
Supplement: Additional file 2: Figure S2 — Secondary structure predictions of aligned snRNAs. [file 1471-2164-14-931-S2.pdf]

FIG. S2

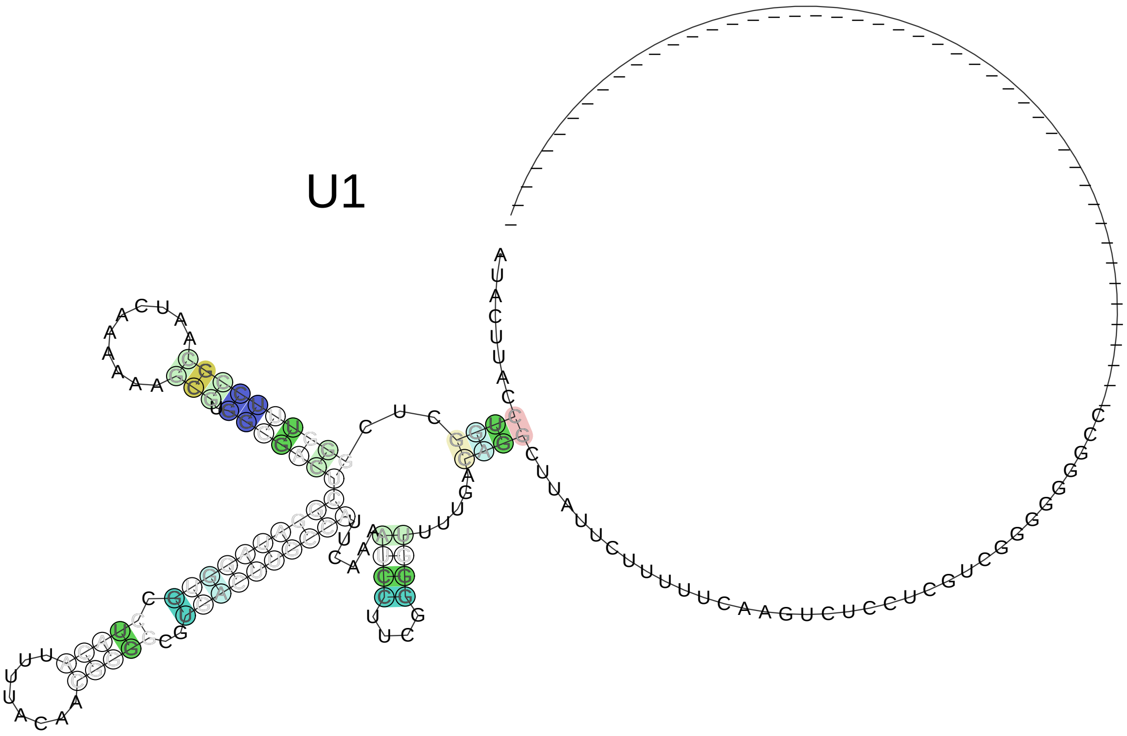


```
T.rubrum        AUACUUACCUUGUUUGGGCUGGCGCGAU-CAAAAAGGC--GCUUCGCCGUGGAAAGGCUC 57
T.equinum       AUACUUACCUUGUUUGGGCUGGCGCGAU-CAAAAAGGC--GCUUCGCCGUGGAAAGGCUC 57
A.fumigatus     AUACUUACCUUGUUUGGGUGGCUGCGAU-CAAGAAGGC--GGUCCACCACGGAUUGGUCC 57
A.niger         AUACUUACCUUGUUUGGGUGACUGCGAU-CAAGAAGGC--GGUUCACCACGGAUUGGUCC 57
R.oryzae        AUACUUACCUGGGUCGUGUAUUCGGAAU-CAAGAAUCCCGAAUAUACAGUGAAAGUGGUU 59
S.pombe         UUACUUACCUGGCAUGAGUUUCUGCAGCACAAGAAUUGUGGAGACUCAGUU-AUUUGUCU 59
S.octosporus    UUACUUACCUGGCACGGGUUUUCGCAGCUCAAGAAUUGUGGGAAUUCAGCU-GUUUGUCU 59
                 ********* *   * *     *     *** **            *         *

T.rubrum        CUACAUUGCAC---AGCG-UGGAGAGCCUUGCCACUCAACAUCCUUCGGGGUUUUGACAG 113
T.equinum       CUACAUUGCAC---AGCG-UGGAGAGCCUUGCCACUCAACAUCCUUCGGGGUUUUGACAG 113
A.fumigatus     CUGCAUUGCAC---AGCG-CGGCGGAACAAUCCGUUCAACGGCCUUCGGGUCAUUGACAG 113
A.niger         CUGCAUUGCAC---AGCG-CGGCGGAACAAUCCGUUCAACGGCCUACGGGUCAUUGACAG 113
R.oryzae        GU-CAUUGCAC---AGCGACCGCUGUUUUUGCUGCG-AACGCUCUUCGGGGCGUCUCGAG 114
S.pombe         UGGCAUUGCACUG-AGCCCUGACGAA--UAACUGUG-GACUGGCUAAGGUCAGCUCCGGA 115
S.octosporus    UUGCAUUGCACUGUAGCUCUGACGGA--CAGUUGCG-GACUGGCUACGGUCAGAUCCGAG 116
                   ********   ***                      **   **  **

T.rubrum        G-CAUAUUCUUUG--------GCACCUCUC-CUGGACU-CAGGUCCUACCUUUCUUUUCA 162
T.equinum       G-CAUAUUCUUUG--------GCACCUCUC-CUGGACUUCGGGUCCUAC----------- 152
A.fumigatus     G-CGUAUUCUUUG--------GCUCUAU-------------------------------- 132
A.niger         G-CGUAUUCUUUG--------GCACUUCUC-CU----UUUGGAGCUU------------- 146
R.oryzae        AUCAUAAUUUUUGUUUUGGGGGUAUUGGUGGUUCGCCGCCGAGGCCCCU----------- 163
S.pombe         UGCAUCAUUUUUG---------AGUUCGUCCCUCA--UUUGGGGCA-------------- 150
S.octosporus    UGCAUCAUUUUUG---------AGUUCGUCUCU-G--UUAAGGAGACA------------ 152
                  * *  * ****

T.rubrum        UUGUCUCUGCGCUUUAUGUGCUGUUGCAAAGAAG 196
T.equinum       ----------------------------------
A.fumigatus     ----------------------------------
A.niger         ----------------------------------
R.oryzae        ----------------------------------
S.pombe         ----------------------------------
S.octosporus    ----------------------------------
```

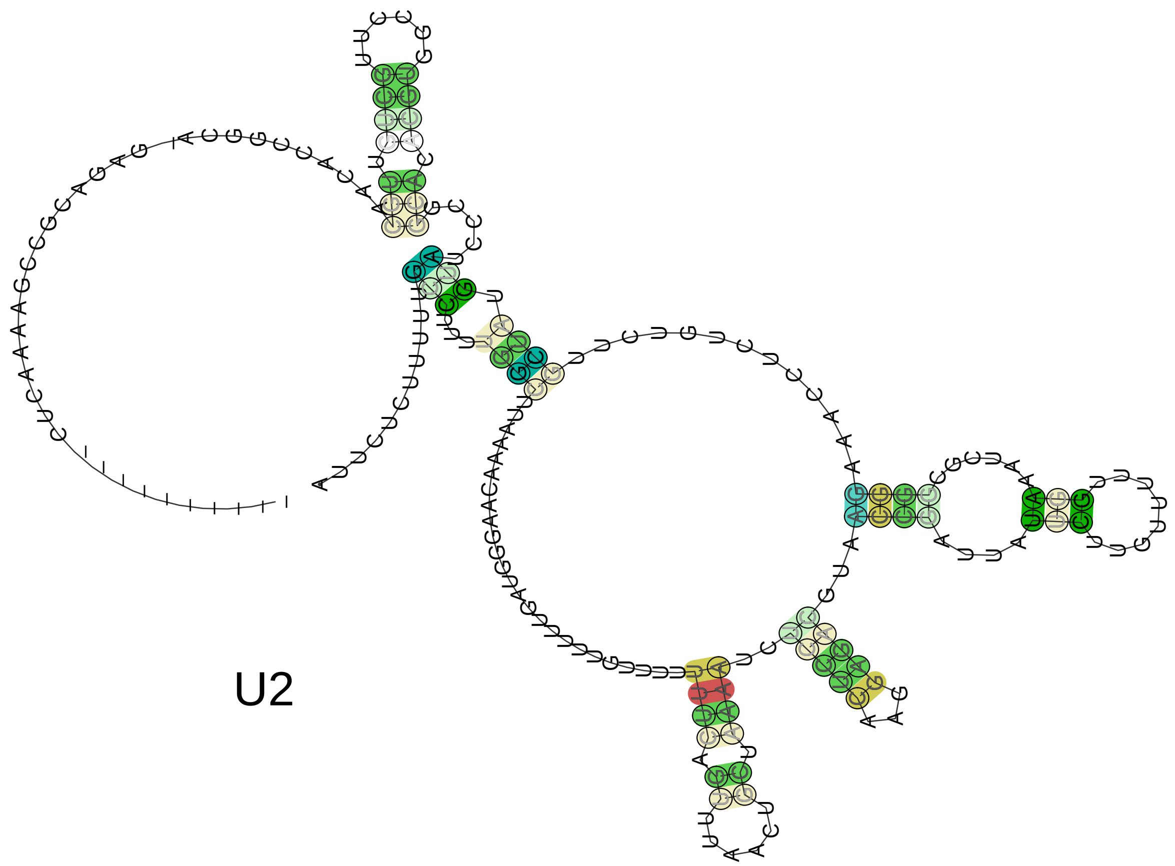

U2

```
T.rubrum          ---GCUCUCUUUGCCAUUAUGGCUUAGAUCAAGUGUAGUAUCUGUUCUUUUCAGUUUAAU 57
A.nidulans        CCAGCUCUCUUUGCCUUU-UGGCUUAGAUCAAGUGUAGUAUCUGUUCUUUUCAGUUUAAU 59
S.octosporus      -AUUCUCUCUUUGCCUUU-UGGCUUAGAUCAAGUGUAGUAUCUGUUCUUUUCAGUUUAAU 58
S.japonicus       -AUUCUCUCUUUGCCUUU-UGGCUUAGAUCAAGUGUAGUAUCUGUUCUUUUCAGUUUAAU 58
S.cerevisiae      ----AUCUCUUUGCCUUU-UGGCUUAGAUCAAGUGUAGUAUCUGUUCUUUUCAGUGUAAC 55
                       *********** ** ************************************ ***

T.rubrum          CUCUGAAAGUAUUCUAAGGAAUACGAAAGUGAUUAUUCUUAUUUUUGUACCGCGCGAUUA 117
A.nidulans        CUCUGAAAGUGUUCUAAGGAACACAACAUUGAUUAAUCUUAUUUUUGGAUCUCGUGGCAG 119
S.octosporus      CGCUGAAAUCGCCUCACUGAG-GUGUUUCCGAUUAAUCUUGUUUUUGGUAUUUGCUGGAA 117
S.japonicus       CGCUGAAAUCACCCCAUCGGG-GUGUUUCCGAUUAUUCUUGUUUUUGGGAAUCGCCGAGA 117
S.cerevisiae      AACUGAAAUGACCUCAAUGAG---------GCUCA---UUACCUUU-UAAUUUGUUACAA 102
                     ******       *  *           * * *   **   ***        *

T.rubrum          GACCUCUGUGCUUGCGCAUGUCUUUUCGCACCG-UGUCAUCGGUCUUACACUACCACCGA 176
A.nidulans        GGCCUCUGUGCUUGCGCAUGCCUUGCCACAUAG-UGUCAUUGGGCUUACACUACCCCCAA 178
S.octosporus      AGCCUCUG-GCUUGC-UAUGUUUUCCGGCACCGGUGUCCUUGCUAUUGCACUACCGGCAA 175
S.japonicus       GACCUAUG-GCUUGC-UAUGUCUCUUGGCACCGGUGUCCUUGCCGUUGCACUCCUGGCAU 175
S.cerevisiae      UAC--------------ACAUUUUUUGGCACC---------------CAAAAUAAUAA-AA 133
                     *              *    *     **                    * *

T.rubrum          UCGACGC-GAACCUACCUCUUUUUUC 201
A.nidulans        UCGACGC-GAACC-----CUU----- 193
S.octosporus      GAGACGCCGAAUC-----AUC----- 191
S.japonicus       GCGACGCCGAAAA-----CUC----- 191
S.cerevisiae      UGGACGG-GAA--------------- 143
                     ****  ***
```

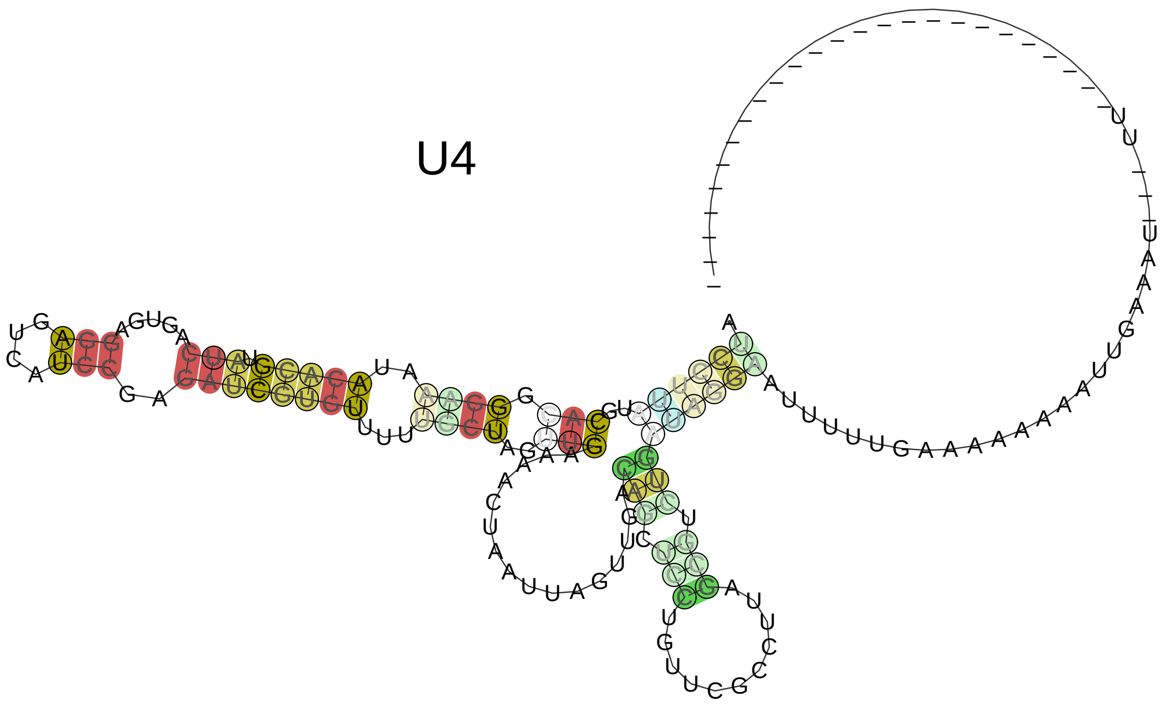


```
A.fumigatus           AUCCUGGUGCACGGGUUUUACACGUGUCAGUGAGGAGACAUCCGAGACCGUGUUUUUUGCU 60
A.oryzae              AUCCUAGUGCACGGGCAAUACACGUAUCAGUGAGGAGACAUCCGAGAUCGUGUUUUUUGCU 60
A.nidulans            AUCCUGGUGCAAGGGUUUUACACGUGUCAGUGAGGAGCCAUCCGAGACCGUGUUUUUUGCU 60
T.rubrum              ----GAGUGCAAGGGUUUUACGUUU-CCAGUGAAGGUUCGCCUGAGGCAACGUUUUUUGCU 55
S.kudriavzevii        AUCCUUAUGCACGGGAAAUACGCAUAUCAGUGAGGAUUCGUCCGAGAUUGUGUUUUUUGCU 60
S.castelliiU4         AUCCUUAUGCAAGGGAAAUACGCAUAUCAGUGAGGAUUCGUCCGAGAUUGUGUUUUUUGCU 60
Candida               GUCUUUAUGCAGGGGCAUUACGUGUAUCAGUGAGGAUUCGUCCGAGAUCAUGUUUUUUGCU 60
                             **** ***    ***    *  ****** *    *  * ***      *********

A.fumigatus           AGUUGAAA----------------ACUACCUAGU--UGACCGCUU--CAG---UUGGUCU 97
A.oryzae              AGUUGAAA----------------ACUAUUUUUU--UGACCGCUC--UAG---UUGGCCU 97
A.nidulans            AGUUGAAA----------------ACUACCUAGU--UGACCGCUC--CAA---UUGGCCU 97
T.rubrum              AGUUGAAA----------------ACUACAAACCAAUGACCGCUC--UAG---UUGGUCU 94
S.kudriavzevii        GGUUGAAAUUUAAUUAUAAACCAGACCGUCUCCUCAUGGUCGCUUAUCGG-UGUUCGCUU 119
S.castelliiU4         GGUUGAAAUUUAAUU--AAACCAGACCUGCUCCUCAUUGUCGCCAGUUAGAUGUUCGCUU 118
Candida               GGUUGAAA---ACUU--AAACCUGAC-AACUUCU--UGAAUGCUA--UAG-UAUUUUACU 109
                       *******                **            *    **            **    *

A.fumigatus           U-CGGA----UCG-------GCUGGGAAUUUUUGAAAAAAAGUUGCCCUGACAUCCUAAG 145
A.oryzae              U-CGGG----UCG-------GCUAGGAAUUUUUGAAAACAAACUGCCCUGGC-UCAUA-- 142
A.nidulans            U-AGGG----UCG-------GUUGGGAAUUUUUGAACA--------CUUAA----AGAAA 133
T.rubrum              U-CGGA----UCG-------GCUGGGAAUUUUUGAAGAA--------------------- 121
S.kudriavzevii        U-UGAAUACUUCAAGACUAUGUAGGGAAUUUUUGGAA----------------------- 155
S.castelliiU4         CAUGAACAUCUCA-GAUAAUGUAGGGAAUUUUUGGAAUACUUUUAUU------------- 164
Candida               UGUGUG---UUUA---------AGAGAAUUUUUGGAACCAUUU----------------- 140
                          *      *              ********* *

A.fumigatus           UCUUGGUGUCAGUUUU 161
A.oryzae              -UUUG----------- 146
A.nidulans            ----------------
T.rubrum              ----------------
S.kudriavzevii        ----------------
S.castelliiU4         ----------------
Candida               ----------------
```

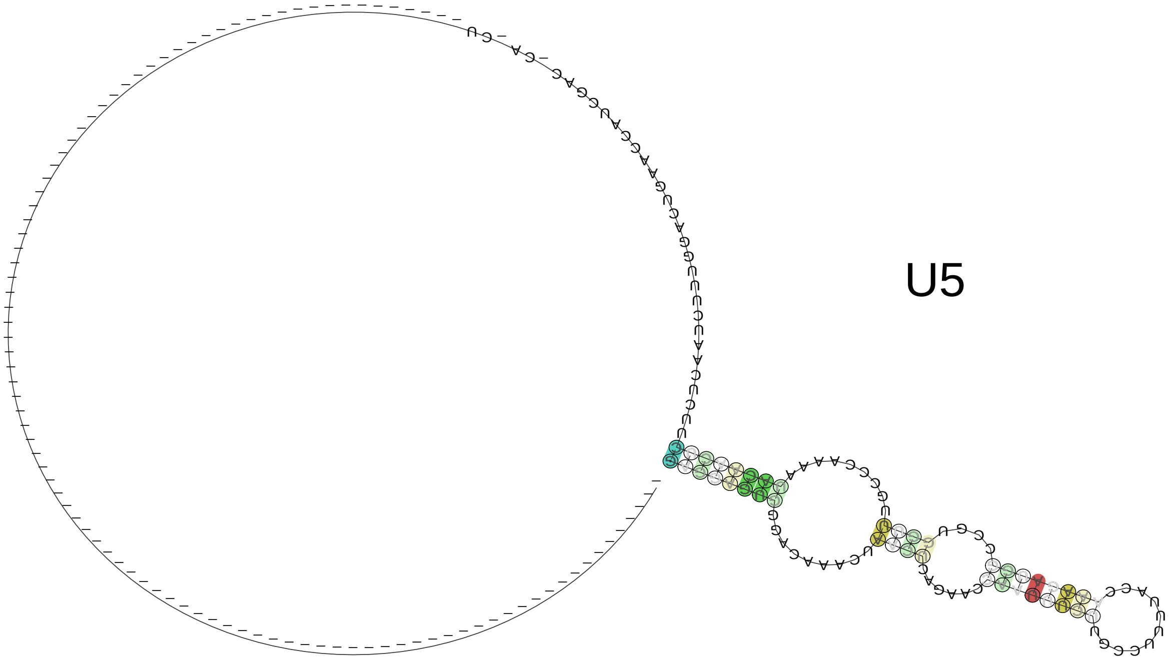

U5

```
S.pombe           -GUCAAAGCACUUUGCAAAAGCUA--AC-GUAUCUGUUUCUUGCCUUUUACCAGAAACAG 56
S.kudriavzevii    -UUUGUUGC---CUACAGAACUUAUAAC-GAACAUGGUUCUUGCCUUUUACCAGAACCAU 55
A.fumigatus       ----GAGGAG--UGGGACAAGCUAGCUCAGAACCCAUUUCUUGCCUUUUACCAGAGAUGG 54
A.clavatus        ----GAGGAG--UGGGACAAGCUAGCUCAGAACCCAUUUCUUGCCUUUUACCAGAGAUGG 54
A.niger           ----GAGGAG--UGGGACAAACUAGCUCAGAACCCAUUUCUUGCCUUUUACCAGAGAUGG 54
T.rubrum          CUGCGAGGAG--UGGGACAAACUAGCUCAGAAUCCAUUUCUUGCCUUUUACCAGAGAUGG 58
R.oryzae          ------UACUUCUGUCUUGUGCUCAUAUCGGAUAAAUCUUUCGCCUUUUACUAAAGAUUU 54
                                         *      * *       * * ********* * *

S.pombe           CCGUUUGUAAGGUGUGCUAAU-UUGACUGU-----AUAGUUUUUGAAUCUUUU--UCUUG 108
S.kudriavzevii    CCGGGUGU----UGUCUCCAU-UAAA---------ACAGGC------------------A 83
A.fumigatus       CCGUG-GC----UU-GCCCAA-CAUACUCCUCUUCUCAAUCUUUGGACCUUU-------- 99
A.clavatus        CCGUG-GC----UU-GCCCAA-AAUACUCCUCUUCUCAAUCUUUGGACUUAUC--CAUCG 105
A.niger           CCGUG-GU----UU-GCCCAA-AAGACUCCUCUCAUCAAUCUUUGGAAUCACC--CAUCG 105
T.rubrum          CCGUG-GU----UUUGCCCAU-AAUACUCCGCUCACCAAUCUUUGAACCUCCGAAUAUCG 112
R.oryzae          CCGUGGAUGCGGACACUAAAGAAGAUUAC----CCGAAUUUUUUGG-----------UA 99
                  ***                 *     *           *

S.pombe           AAACAU---------- 114
S.kudriavzevii    AAGC------------ 87
A.fumigatus       ----------------
A.clavatus        ACACAGCUGUCGAUU- 120
A.niger           ACACAGCUGUC----- 116
T.rubrum          ACA------------- 115
R.oryzae          AAACCCUCUCUUUUUC 115
```

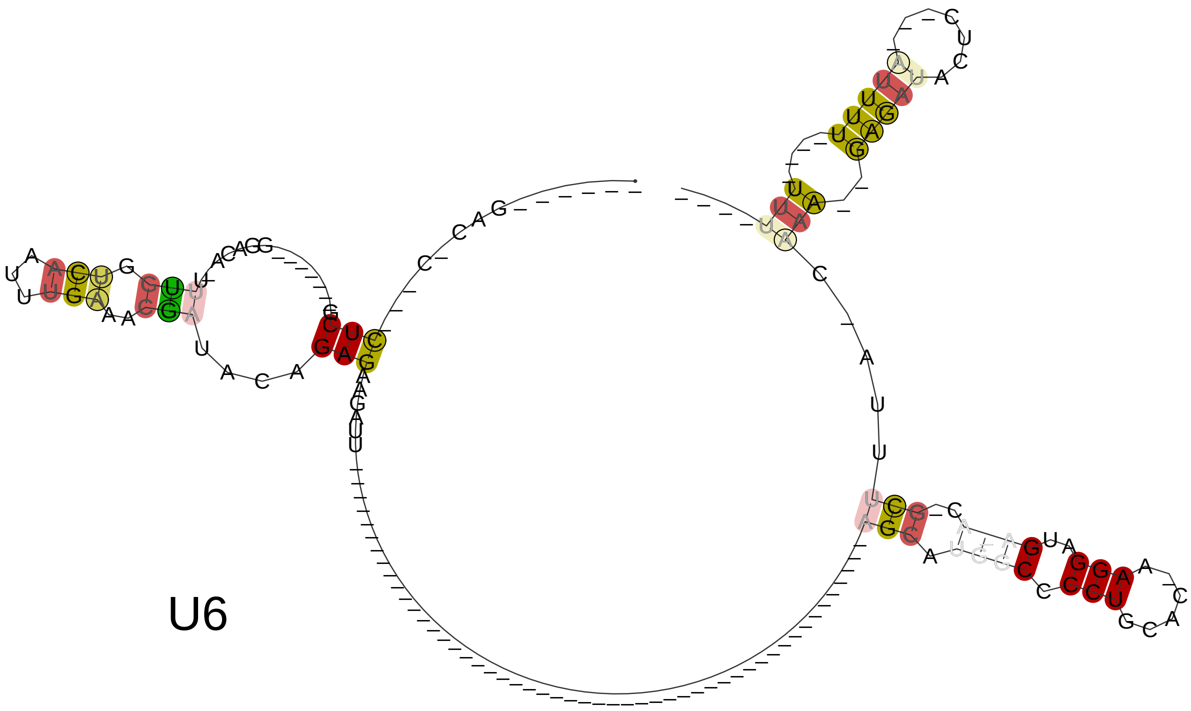

U6

```
S.castellii      GGC---AACCC---CUCG------UGGACA-UUUGGUCAAUUUGAAACAAUACAGAGAUG 47
S.kluyveri       GUCCAAGACAU---UUCGGUGUUUUGGACA-UUUGGUCAAUUUGAAACAAUACAGAGAUG 56
T.rubrum         -------GC-C---UUCG--------GGCA-UUUGGUCA--UAGUAACGAUACAGAGAAG 38
A.oryzae         -------GC-C---CUCG-------AGGCA-UCCGGUCAAUUUGAAACGAUACAGAGAAG 41
S.pombe          -----UGAU-C---UUCG-------GAUCACUUUGGUCAAAUUGAAACGAUACAGAGAAG 44
A.niger          ----UGGACAUAUGCUCG---------ACA---UUGCUAAAUUGGAACGAUACAGAGACG 44
                                ***          **      *  *  * * *** ********* *

S.castellii      AUC--------------------------------------------------AGCAGUU 57
S.kluyveri       AUC--------------------------------------------------AGCAGUU 66
T.rubrum         AUU--------------------------------------------------AGCAUGG 48
A.oryzae         AUU--------------------------------------------------AGCAUGG 51
S.pombe          AUUGUAAGUAACAAUAUUUACCAAGGUUCGAGUCAUACUAACUCGUUGUUUAGAGCAUGG 104
A.niger          AU---------------------------------------------------AGCAUGU 53
                 **                                                   ****

S.castellii      CCCCUGCAU-AAGGAUGA-ACCGUUUUA-CAAA--GAGAUUUA-UUCGUUUU----UU-- 105
S.kluyveri       CCCCUGCAU-AAGGAUGA-ACCGUUUUA-CAAA--GAGAUUUACUUCAUUUU----U--- 114
T.rubrum         CCCCUGCACUAAGGAUGACAC-GCUCAAUCAAA--GAGAAGCUACCAGUUUU----UUUU 101
A.oryzae         CCCCUGCAC-AAGGUUGACAC-GCUUUAUCAAG--GAGAUGCUA--AACUUU----UUU- 100
S.pombe          CCCCUGCAC-AAGGAUGACACUGCGACAUUGAG--AGAAAACCC---AUUUU----UUU- 153
A.niger          CCCCUGCGC-AAGGAUGA-----CGUUA-CAAAUCGAGAUAUUC---GUUUUAGGCUUUU 103
                 *******   **** ***          *   *      *          ***    *

S.castellii      ---
S.kluyveri       ---
T.rubrum         UGG 104
A.oryzae         ---
S.pombe          ---
A.niger          ---
```

FIG. S2 Secondary structures prediction of aligned snRNAs.

T. rubrum snRNAs are compared with the homologs in other fungi using the multiple sequence alignment software Clustal W2. The secondary structures of aligned sequences are predicted by RNAalifold [27, 28]. The color of the bases changes from black to grey if one or more sequences in the alignments deviate from the consensus. If the homologous sequences shows a gap in the alignment, the gap character (-) will be signed in the secondary structures graphs. The results show that these snRNAs are with conserved regions in the hairpin loops.
