# Supplementary material for: Identification and characterisation of non-coding small RNAs in the pathogenic filamentous fungus Trichophyton rubrum
Source: BMC Genomics. 2013 Dec 30;14:931. doi: 10.1186/1471-2164-14-931 (PMC3890542; doi:10.1186/1471-2164-14-931)
Supplement: Additional file 3: Figure S3 — Potential base-paring between H/ACA box snoRNAs and rRNAs predicted by snoGPS. [file 1471-2164-14-931-S3.pdf]

S3. Potential base-paring between H/ACA box snoRNAs and rRNAs predicated by snoGPS. Predicated peudordylation sites are denoted by Ψ.

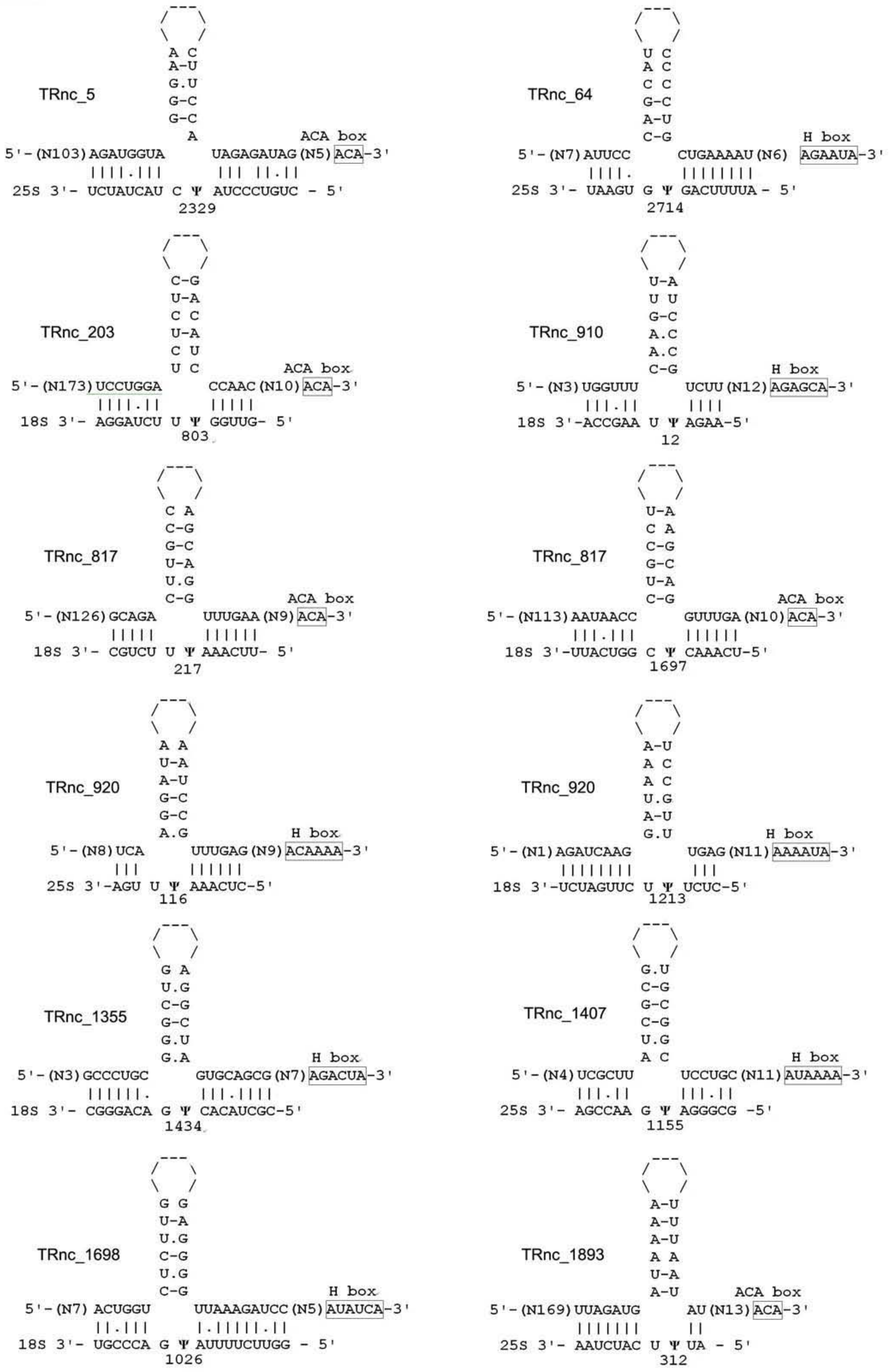

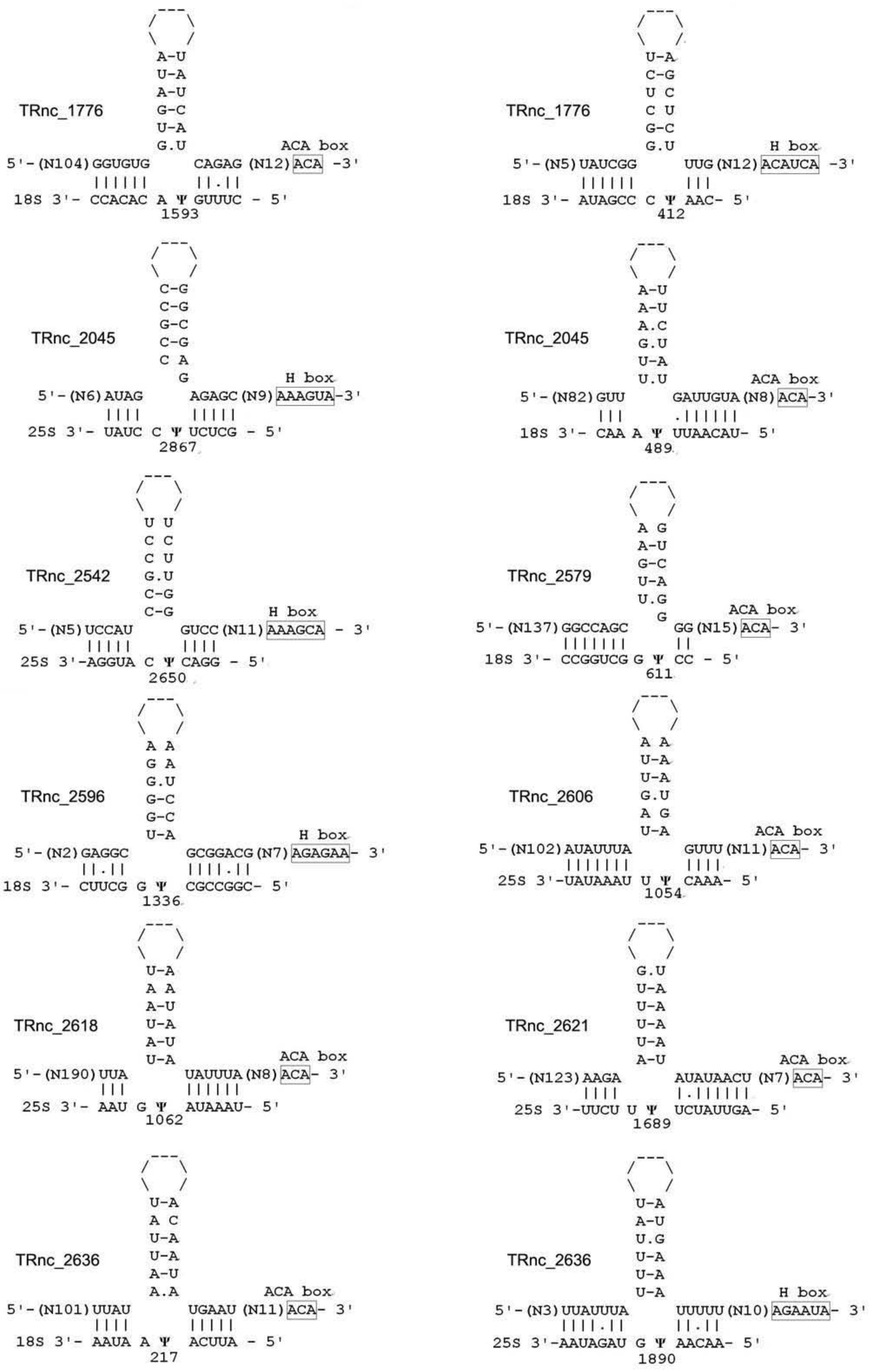

TRnc_1776
A-U
U-A
A-U
G-C
U-A
G.U
ACA box
5'-(N104) GGUGUG CAGAG (N12) ACA -3'
18S 3'- CCACAC A Ψ GUUUC - 5'
1593
TRnc_1776
U-A
C-G
U C
C U
G-C
G.U
H box
5'-(N5) UAUCGG UUG (N12) ACAUCA -3'
18S 3'- AUAGCC C Ψ AAC- 5'
412
TRnc_2045
C-G
C-G
G-C
C-G
C A
G
H box
5'-(N6) AUAG AGAGC (N9) AAAGUA-3'
25S 3'- UAUC C Ψ UCUCG - 5'
2867
TRnc_2045
A-U
A-U
A.C
G.U
U-A
U.U
ACA box
5'-(N82) GUU GAUUGUA (N8) ACA-3'
18S 3'- CAA A Ψ UUAACAU- 5'
489
TRnc_2542
U U
C C
C U
G.U
C-G
C-G
H box
5'-(N5) UCCAU GUCC (N11) AAAGCA - 3'
25S 3'-AGGUA C Ψ CAGG - 5'
2650
TRnc_2579
A G
A-U
G-C
U-A
U.G
G
ACA box
5'-(N137) GGCCAGC GG (N15) ACA- 3'
18S 3'- CCGGUCG G Ψ CC - 5'
611
TRnc_2596
A A
G A
G.U
G-C
G-C
U-A
H box
5'-(N2) GAGGC GCGGACG (N7) AGAGAA- 3'
18S 3'- CUUCG G Ψ CGCCGGC- 5'
1336
TRnc_2606
A A
U-A
U-A
G.U
A G
U-A
ACA box
5'-(N102) AUAUUUA GUUU (N11) ACA- 3'
25S 3'-UAUAAAU U Ψ CAAA- 5'
1054
TRnc_2618
U-A
A A
A-U
U-A
A-U
U-A
ACA box
5'-(N190) UUA UAUUUA (N8) ACA- 3'
25S 3'- AAU G Ψ AUAAAU- 5'
1062
TRnc_2621
G.U
U-A
U-A
U-A
U-A
A-U
ACA box
5'-(N123) AAGA AUAUAACU (N7) ACA- 3'
25S 3'-UUCU U Ψ UCUAUUGA- 5'
1689
TRnc_2636
U-A
A C
U-A
U-A
A-U
A.A
ACA box
5'-(N101) UUAU UGAAU (N11) ACA- 3'
18S 3'- AAUA A Ψ ACUUA - 5'
217
TRnc_2636
U-A
A-U
U.G
U-A
U-A
U-A
H box
5'-(N3) UUAUUUA UUUUU (N10) AGAAUA- 3'
25S 3'-AAUAGAU G Ψ AACAA- 5'
1890

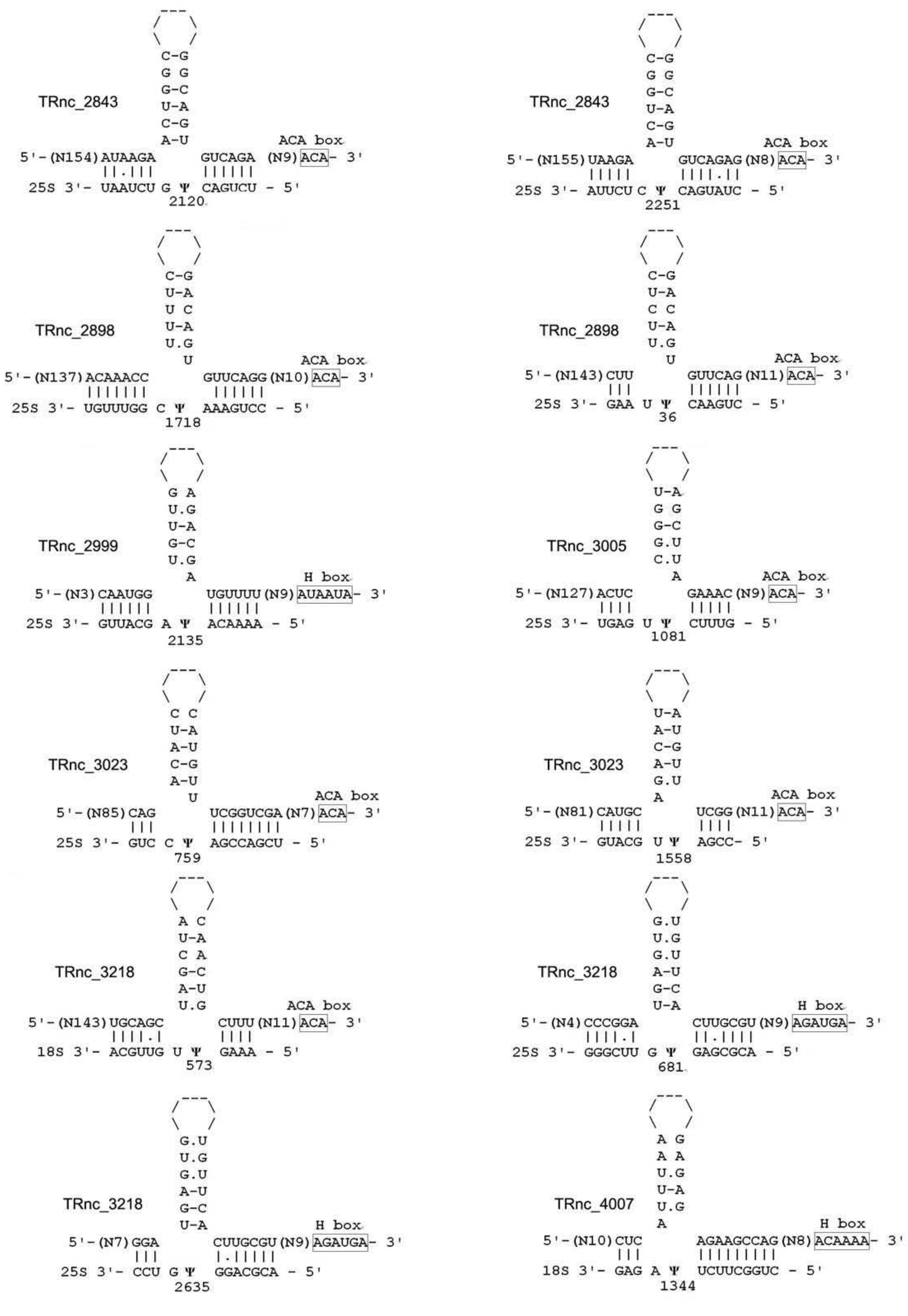

TRnc_2843
C-G
G G
G-C
U-A
C-G
A-U
ACA box
5'-(N154) AUAAGA GUCAGA (N9) ACA- 3'
25S 3'- UAAUCU G Ψ CAGUCU - 5'
2120
TRnc_2843
C-G
G G
G-C
U-A
C-G
A-U
ACA box
5'-(N155) UAAGA GUCAGAG (N8) ACA- 3'
25S 3'- AUUCU C Ψ CAGUAUC - 5'
2251
TRnc_2898
C-G
U-A
U C
U-A
U.G
U
ACA box
5'-(N137) ACAAACC GUUCAGG (N10) ACA- 3'
25S 3'- UGUUUGG C Ψ AAAGUCC - 5'
1718
TRnc_2898
C-G
U-A
C C
U-A
U.G
U
ACA box
5'-(N143) CUU GUUCAG (N11) ACA- 3'
25S 3'- GAA U Ψ CAAGUC - 5'
36
TRnc_2999
G A
U.G
U-A
G-C
U.G
A
H box
5'-(N3) CAAUGG UGUUUU (N9) AUAAUA- 3'
25S 3'- GUUACG A Ψ ACAAAA - 5'
2135
TRnc_3005
U-A
G G
G-C
G.U
C.U
A
ACA box
5'-(N127) ACUC GAAAC (N9) ACA- 3'
25S 3'- UGAG U Ψ CUUUG - 5'
1081
TRnc_3023
C C
U-A
A-U
C-G
A-U
U
ACA box
5'-(N85) CAG UCGGUCGA (N7) ACA- 3'
25S 3'- GUC C Ψ AGCCAGCU - 5'
759
TRnc_3023
U-A
A-U
C-G
A-U
G.U
A
ACA box
5'-(N81) CAUGC UCGG (N11) ACA- 3'
25S 3'- GUACG U Ψ AGCC- 5'
1558
TRnc_3218
A C
U-A
C A
G-C
A-U
U.G
ACA box
5'-(N143) UGCAGC CUUU (N11) ACA- 3'
18S 3'- ACGUUG U Ψ GAAA - 5'
573
TRnc_3218
G.U
U.G
G.U
A-U
G-C
U-A
H box
5'-(N4) CCCGGA CUUGCGU (N9) AGAUGA- 3'
25S 3'- GGGCUU G Ψ GAGCGCA - 5'
681
TRnc_3218
G.U
U.G
G.U
A-U
G-C
U-A
H box
5'-(N7) GGA CUUGCGU (N9) AGAUGA- 3'
25S 3'- CCU G Ψ GGACGCA - 5'
2635
TRnc_4007
A G
A A
U.G
U-A
U.G
A
H box
5'-(N10) CUC AGAAGCCAG (N8) ACAAAA- 3'
18S 3'- GAG A Ψ UCUUCGGUC - 5'
1344

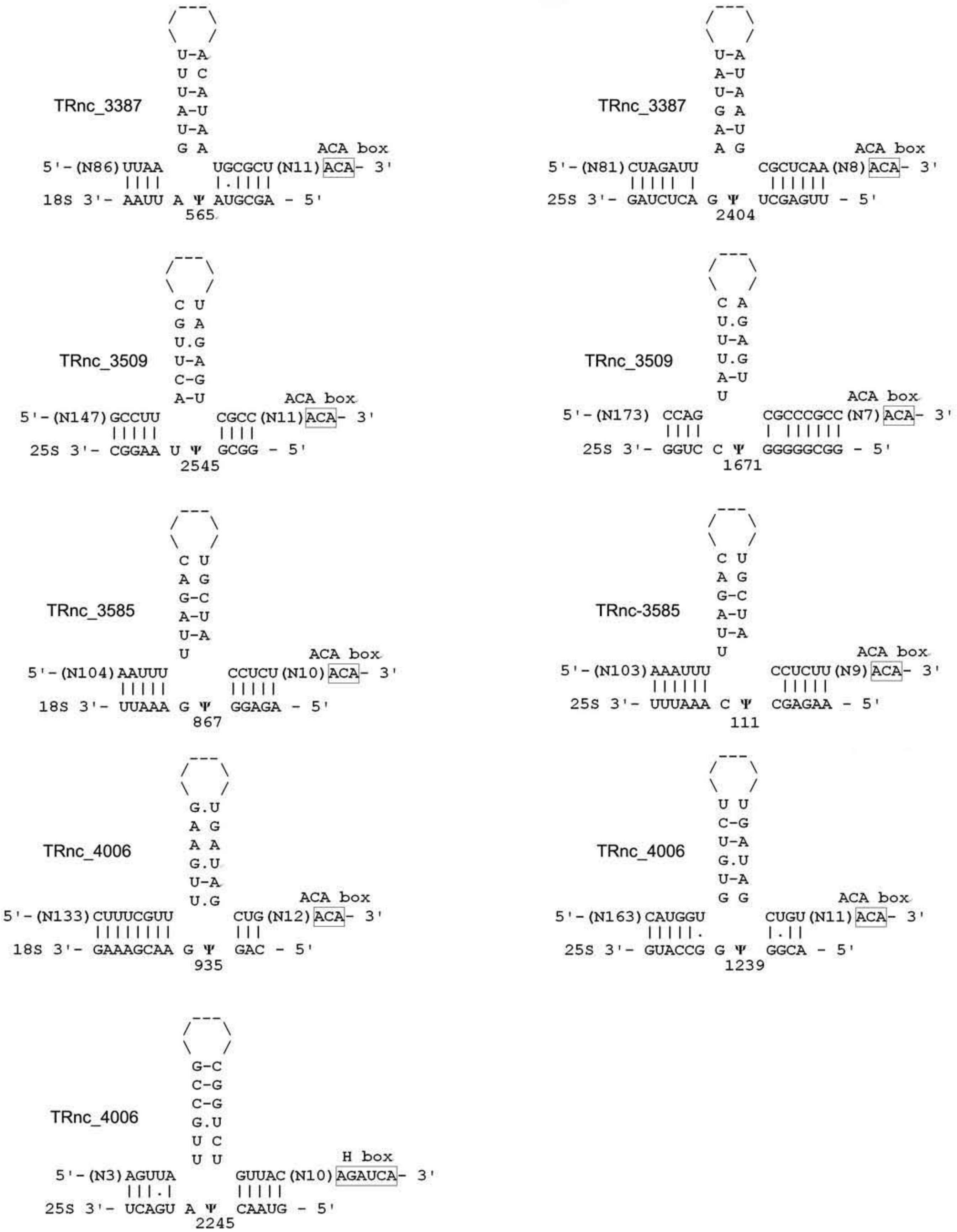
TRnc_3387
U-A
U C
U-A
A-U
U-A
G A
ACA box
5'-(N86) UUAA UGCGCU (N11) ACA- 3'
18S 3'- AAUU A Ψ AUGCGA - 5'
565
TRnc_3387
U-A
A-U
U-A
G A
A-U
A G
ACA box
5'-(N81) CUAGAUU CGCUCAA (N8) ACA- 3'
25S 3'- GAUCUCA G Ψ UCGAGUU - 5'
2404
TRnc_3509
C U
G A
U.G
U-A
C-G
A-U
ACA box
5'-(N147) GCCUU CGCC (N11) ACA- 3'
25S 3'- CGGAA U Ψ GCGG - 5'
2545
TRnc_3509
C A
U.G
U-A
U.G
A-U
U
ACA box
5'-(N173) CCAG CGCCCGCC (N7) ACA- 3'
25S 3'- GGUC C Ψ GGGGGCGG - 5'
1671
TRnc_3585
C U
A G
G-C
A-U
U-A
U
ACA box
5'-(N104) AAUUU CCUCU (N10) ACA- 3'
18S 3'- UUAAA G Ψ GGAGA - 5'
867
TRnc-3585
C U
A G
G-C
A-U
U-A
U
ACA box
5'-(N103) AAAUUU CCUCUU (N9) ACA- 3'
25S 3'- UUUAAA C Ψ CGAGAA - 5'
111
TRnc_4006
G.U
A G
A A
G.U
U-A
U.G
ACA box
5'-(N133) CUUUCGUU CUG (N12) ACA- 3'
18S 3'- GAAAGCAA G Ψ GAC - 5'
935
TRnc_4006
U U
C-G
U-A
G.U
U-A
G G
ACA box
5'-(N163) CAUGGU CUGU (N11) ACA- 3'
25S 3'- GUACCG G Ψ GGCA - 5'
1239
TRnc_4006
G-C
C-G
C-G
G.U
U C
U U
H box
5'-(N3) AGUUA GUUAC (N10) AGAUCA- 3'
25S 3'- UCAGU A Ψ CAAUG - 5'
2245
