# Supplementary material for: Identification and characterisation of non-coding small RNAs in the pathogenic filamentous fungus Trichophyton rubrum
Source: BMC Genomics. 2013 Dec 30;14:931. doi: 10.1186/1471-2164-14-931 (PMC3890542; doi:10.1186/1471-2164-14-931)
Supplement: Additional file 4: Table S4 — Conversed sncRNAs in all seven dermatophytes. [file 1471-2164-14-931-S4.pdf]

S4. Conversed sncRNAs in all seven dermatophytes

Name	Classes	Len (nt)	Reads	Genome Location				Hmologos name in Rfam	
				Chromosome	start	end	position	Accession	Name
Trnc_293	ncRNA	116	917	supercont2.1	1477742	1477627	3′UTR	no	no
Trnc_305	ncRNA	97	579	supercont2.1	1515685	1515781	Intron	no	no
Trnc_500	ncRNA	203	1	supercont2.1	2298649	2298447	Intron	no	no
Trnc_611	ncRNA	104	1	supercont2.1	2710988	2711091	Intron	no	no
Trnc_718	ncRNA	122	1	supercont2.1	3334692	3334571	Intron	no	no
Trnc_733	ncRNA	174	1	supercont2.1	3371115	3371288	3′UTR	no	no
Trnc_789	ncRNA	248	1	supercont2.1	3618013	3618260	5′UTR	no	no
Trnc_155	ncRNA; SscA RNA	102	1	supercont2.1	749414	749313	Intron	RF00063	SscA
Trnc_161	ncRNA;pri- or pre-miRNA	91	1	supercont2.1	792780	792870	5′UTR	no	mir-574
Trnc_112	ncRNA;pri- or pre-miRNA	100	2	supercont2.1	553233	553134	3′UTR	RF01059	mir-598
Trnc_512	ncRNA;pri- or pre-miRNA	195	1	supercont2.1	2323310	2323504	Intron	no	mir-598
Trnc_608	snoRNA;CD-box	234	1	supercont2.1	2701229	2701462	3′UTR	RF01202	sn2991
Trnc_640	snoRNA;CD-box	129	2	supercont2.1	2869815	2869687	3′UTR	RF00300	snoZ221
Trnc_338	snoRNA;CD-box	135	44	supercont2.1	1643180	1643314	Intron	RF01223	snR13
Trnc_415	snoRNA;CD-box	103	1	supercont2.1	1918108	1918210	Intron	RF01121	Sr38
Trnc_801	snoRNA;CD-box	488	919	supercont2.1	3681448	3681935	3′UTR	RF00012	U3
Trnc_817	snoRNA;HACA-box	188	323	supercont2.1	3719705	3719892	3′UTR	no	no
Trnc_774	snRNA;splicing	201	163	supercont2.1	3545014	3545214	3′UTR	RF00004	U2
Trnc_681	snRNA;splicing	211	15583	supercont2.1	3061687	3061897	5′UTR	RF00020	U5
Trnc_877	ncRNA	97	2	supercont2.10	76955	77051	3′UTR	no	no
Trnc_956	ncRNA	241	2	supercont2.10	559285	559525	5′UTR	no	no
Trnc_1008	ribozyme	419	41	supercont2.10	748640	749058	3′UTR	RF00030	RNase_MRP
Trnc_1010	snoRNA;CD-box	87	80	supercont2.10	749220	749306	3′UTR	RF00477	snosnR66
Trnc_1167	ncRNA	177	1	supercont2.11	544895	545071	3′UTR	no	no
Trnc_1211	ncRNA	165	1	supercont2.11	718257	718093	Intron	no	no
Trnc_1344	ncRNA	289	1	supercont2.13	260355	260643	3′UTR	no	no
Trnc_1299	snoRNA;CD-box	109	1	supercont2.13	24837	24729	Intron	RF00593	snoU83B
Trnc_1357	ribozyme	208	1	supercont2.14	153790	153997	5′UTR	RF00621	CoTC_ribozyme
Trnc_1359	snoRNA;CD-box	97	290	supercont2.14	159345	159441	Intron	RF00475	snosnR69
Trnc_1415	ncRNA	140	2	supercont2.16	82202	82063	5′UTR	no	no
Trnc_1423	ncRNA	305	3	supercont2.16	86158	86462	3′UTR	no	no
Trnc_1686	ncRNA	133	126	supercont2.2	1303987	1304119	3′UTR	no	no
Trnc_1702	ncRNA	375	2	supercont2.2	1373166	1372792	3′UTR	no	no
Trnc_1801	ncRNA	209	2	supercont2.2	1873103	1873311	Intron	no	no
Trnc_1792	ncRNA	251	1	supercont2.2	1856556	1856806	Intron	no	no
Trnc_1808	ncRNA	283	2	supercont2.2	1884802	1885084	Intron	no	no
Trnc_1983	ncRNA;pri- or pre-miRNA	86	2	supercont2.2	2887537	2887452	3′UTR	RF01059	mir-598
Trnc_1825	snoRNA;CD-box	309	200	supercont2.2	1958330	1958022	3′UTR	no	no
Trnc_1841	snoRNA;CD-box	143	1	supercont2.2	2090171	2090313	3′UTR	RF01144	sR17
Trnc_1560	snoRNA;CD-box	77	1	supercont2.2	546818	546894	3′UTR	RF01139	sR2
Trnc_1893	snoRNA;HACA-box	344	21	supercont2.2	2393882	2393539	3′UTR	no	no
Trnc_1472	snoRNA;HACA-box	188	170	supercont2.2	69663	69850	3′UTR	RF01258	snR10
Trnc_1782	snRNA;splicing	104	9034	supercont2.2	1801544	1801647	3′UTR	RF00026	U6
Trnc_2354	ncRNA	152	1	supercont2.3	1735194	1735043	Intron	no	no
Trnc_2431	ncRNA	166	1	supercont2.3	2070250	2070085	3′UTR	no	no
Trnc_2448	ncRNA	161	1	supercont2.3	2123075	2122915	5′UTR	no	no
Trnc_2100	ncRNA	134	1	supercont2.3	492158	492291	Intron	no	no
Trnc_2438	ncRNA	460	3	supercont2.3	2078096	2078555	5′UTR	no	no
Trnc_2526	ncRNA	321	288	supercont2.3	2582456	2582136	3′UTR	no	no
Trnc_2565	ncRNA; Cis-reg	102	1	supercont2.3	2757753	2757652	Intron	RF01068	mini-ykkC
Trnc_2163	ncRNA;pri- or pre-miRNA	137	4	supercont2.3	874758	874894	Intron	RF00647	mir164

Trnc_2179	snoRNA;CD-box	431	1	supercont2.3	961995	961565	Intergenic	no	no
Trnc_2405	snoRNA;CD-box	317	1	supercont2.3	1975149	1975465	3′UTR	no	no
Trnc_2265	snoRNA;CD-box	87	187	supercont2.3	1276133	1276219	Intron	RF01197	snR39
Trnc_2545	snoRNA;CD-box	119	27	supercont2.3	2657688	2657806	3′UTR	RF01188	snR56
Trnc_2594	snoRNA;CD-box	143	2	supercont2.3	2859175	2859033	Intron	RF01305	sR51
Trnc_2452	snoRNA;HACA-box	323	1	supercont2.3	2170039	2169717	3′UTR	no	no
Trnc_2172	snoRNA;HACA-box	126	1	supercont2.3	922150	922025	Intron	RF00406	SNORA42
Trnc_2075	snoRNA;HACA-box	96	5	supercont2.3	425677	425772	Intron	RF00405	SNORA44
Trnc_2676	ncRNA	156	2	supercont2.4	110438	110593	3′UTR	no	no
Trnc_2821	ncRNA	268	1	supercont2.4	837487	837220	5′UTR	no	no
Trnc_2839	ncRNA	186	11647	supercont2.4	948628	948443	5′UTR	no	no
Trnc_2844	ncRNA	239	225	supercont2.4	981025	981263	5′UTR	no	no
Trnc_2704	ncRNA	80	1	supercont2.4	350218	350297	Intron	no	no
Trnc_2966	ncRNA	103	1	supercont2.4	1562900	1562798	3′UTR	no	no
Trnc_2935	ncRNA; Cis-reg	162	9	supercont2.4	1403494	1403333	Intron	RF00220	Rhino_CRE
Trnc_2936	snoRNA;CD-box	246	16	supercont2.4	1403883	1403638	Intron	RF00312	snoZ206
Trnc_2898	snoRNA;HACA-box	393	1	supercont2.4	1199167	1198775	Intron	no	no
Trnc_2843	snoRNA;HACA-box	225	56	supercont2.4	976176	976400	3′UTR	RF01251	snR3
Trnc_3157	ncRNA	177	1	supercont2.5	328773	328597	Intron	no	no
Trnc_3176	ncRNA	137	1	supercont2.5	423569	423705	3′UTR	no	no
Trnc_3179	ncRNA	207	39	supercont2.5	431925	431719	3′UTR	no	no
Trnc_3237	ncRNA	164	673	supercont2.5	722162	721999	Intron	no	no
Trnc_3392	ncRNA	180	1	supercont2.5	1543669	1543848	Intron	no	no
Trnc_3269	ncRNA; Cis-reg	121	1	supercont2.5	832731	832611	3′UTR	RF00180	REN-SRE
Trnc_3297	snoRNA;CD-box	138	12	supercont2.5	896392	896529	3′UTR	RF00610	SNORD110
Trnc_3569	ncRNA;pri- or pre-miRNA	297	1	supercont2.6	934438	934142	Intron	RF01059	mir-598
Trnc_3425	snoRNA;CD-box	202	94	supercont2.6	22581	22782	3′UTR	no	no
Trnc_3426	snoRNA;CD-box	98	761	supercont2.6	23000	23097	3′UTR	no	no
Trnc_3573	snoRNA;CD-box	95	147	supercont2.6	964586	964680	Intron	RF00530	[illegible]oMe28S-Cm264
Trnc_3509	snoRNA;HACA-box	433	1	supercont2.6	608530	608098	5′UTR	no	no
Trnc_3585	snoRNA;HACA-box	281	1	supercont2.6	1065274	1064994	Intron	no	no
Trnc_3841	ncRNA	209	1	supercont2.7	1169120	1169328	3′UTR	no	no
Trnc_3725	ncRNA; Cis-reg	139	1	supercont2.7	440095	439957	3′UTR	RF01088	TLS-PK5
Trnc_3783	ncRNA;pri- or pre-miRNA	80	1	supercont2.7	784466	784545	3′UTR	RF00990	mir-552
Trnc_3778	snoRNA;CD-box	101	31	supercont2.7	777627	777727	5′UTR	RF00471	snosnR48
Trnc_3741	snoRNA;HACA-box	180	42	supercont2.7	491853	492032	3′UTR	RF01247	snR32
Trnc_4066	ncRNA	230	2	supercont2.8	925945	926174	3′UTR	no	no
Trnc_3999	ncRNA	178	5	supercont2.8	672734	672557	3′UTR	no	no
Trnc_3959	no	82	1	supercont2.8	495724	495805	3′UTR	no	no
Trnc_3911	snoRNA;CD-box	80	3	supercont2.8	194694	194773	Intron	RF00213	snoR38
Trnc_4113	snoRNA;CD-box	681	22	supercont2.8	1152047	1152727	Intron	RF01274	sR45
Trnc_4006	snoRNA;HACA-box	251	28	supercont2.8	710950	711200	Intron	RF01263	snR191
Trnc_3904	snRNA;splicing	196	1238	supercont2.8	159538	159733	5′UTR	RF00003	U1
Trnc_4219	ncRNA	104	1	supercont2.9	449429	449532	5′UTR	no	no
Trnc_4267	snoRNA;CD-box	100	10	supercont2.9	703004	703103	3′UTR	no	no
Trnc_4260	snoRNA;CD-box	95	163	supercont2.9	695194	695288	Intergenic	RF01178	snoR77Y
Trnc_4263	snoRNA;CD-box	157	486	supercont2.9	695917	696073	Intergenic	RF00086	SNORD27
Trnc_4259	snoRNA;CD-box	104	892	supercont2.9	693331	693434	5′UTR	RF00276	SNORD52
Trnc_4264	snoRNA;CD-box	88	23	supercont2.9	696179	696266	5′UTR	RF01207	snR73
Trnc_4262	snoRNA;CD-box	273	603	supercont2.9	695588	695860	Intergenic	RF01185	snR75
Trnc_4261	snoRNA;CD-box	138	5548	supercont2.9	695445	695582	Intergenic	RF01209	snR76
